# Refining the Identity and Role of Kv4 Channels in Mouse Substantia Nigra Dopaminergic Neurons

**DOI:** 10.1523/ENEURO.0207-21.2021

**Published:** 2021-07-20

**Authors:** Alexis Haddjeri-Hopkins, Mónica Tapia, Jorge Ramirez-Franco, Fabien Tell, Béatrice Marqueze-Pouey, Marianne Amalric, Jean-Marc Goaillard

**Affiliations:** 1 Unité Mixte de Recherche 1072, Aix Marseille University, Institut National de la Santé et de la Recherche Médicale, Faculté de Médecine Secteur Nord, Marseille 13015, FRANCE; 2 Unité Mixte de Recherche 7291, Aix Marseille University, Centre National de la Recherche Scientifique, Laboratoire de Neurosciences Cognitives, Marseille 13331, France

**Keywords:** biophysics, computational modeling, dopamine, patch clamp, potassium channels, substantia nigra

## Abstract

Substantia nigra pars compacta (SNc) dopaminergic (DA) neurons display a peculiar electrical phenotype characterized *in vitro* by a spontaneous tonic regular activity (pacemaking activity), a broad action potential (AP) and a biphasic postinhibitory response. The transient A-type current (I_A_) is known to play a crucial role in this electrical phenotype, and so far, this current was considered to be carried exclusively by Kv4.3 potassium channels. Using Kv4.3−/− transgenic mice, we demonstrate that the constitutive loss of this channel is associated with increased exploratory behavior and impaired motor learning at the behavioral level. Consistently, it is also associated with a lack of compensatory changes in other ion currents at the cellular level. Using antigen retrieval (AR) immunohistochemistry, we then demonstrate that Kv4.2 potassium channels are also expressed in SNc DA neurons, although their contribution to I_A_ appears significant only in a minority of neurons (∼5–10%). Using correlative analysis on recorded electrophysiological parameters and multicompartment modeling, we then demonstrate that, rather than its conductance level, I_A_ gating kinetics (inactivation time constant) appear as the main biophysical property defining postinhibitory rebound delay and pacemaking frequency. Moreover, we show that the hyperpolarization-activated current (I_H_) has an opposing and complementary influence on the same firing features.

## Significance Statement

Substantia nigra pars compacta (SNc) dopaminergic (DA) neurons are characterized by pacemaking activity, a broad action potential (AP) and biphasic postinhibitory response. The A-type transient potassium current (I_A_) plays a central role in this electrical phenotype. While it was thought so far that Kv4.3 ion channels were fully responsible for I_A_, using a Kv4.3−/− transgenic mouse and antigen retrieval (AR) immunohistochemistry we demonstrate that Kv4.2 channels are also expressed in SNc DA neurons, although their contribution is significant in a minority of neurons only. Using electrophysiological recordings and computational modeling, we then demonstrate that I_A_ gating kinetics and its functional complementarity with the hyperpolarization-activated current are major determinants of both pacemaking activity and postinhibitory response in SNc DA neurons.

## Introduction

While the expression of only two types of voltage-gated ion channels in the squid giant axon allowed Hodgkin and Huxley to dissect the biophysical processes underlying action potential (AP) genesis and conduction (Hodgkin and Huxley, 1952), most neuronal types express a multitude of ion channel subtypes underlying their electrical activity (Cembrowski et al., 2016; Fuzik et al., 2016; Tapia et al., 2018; Northcutt et al., 2019). In spontaneously active neurons, a variety of voltage-gated and calcium-gated ion channels are not only responsible for the AP, but also govern the subthreshold oscillations leading to AP firing, determine firing frequency and control its regularity (Atherton and Bevan, 2005; Swensen and Bean, 2005; Bean, 2007; Gantz et al., 2018). Substantia nigra pars compacta (SNc) dopaminergic (DA) neurons spontaneously generate a regular tonic pattern of activity, also known as “pacemaking” activity (Grace and Onn, 1989; Gantz et al., 2018). Over the past 40 years, many studies contributed to the identification of the specific ion channels involved in shaping pacemaking activity (Nedergaard and Greenfield, 1992; Liss et al., 2001, 2005; Seutin et al., 2001; Wolfart et al., 2001; Neuhoff et al., 2002; Chan et al., 2007; Puopolo et al., 2007; Guzman et al., 2009; Putzier et al., 2009a; Ji et al., 2012; Gantz et al., 2018). In particular, several studies have suggested that the transient A-type potassium current (I_A_) plays an essential role in controlling pacemaking rate and postinhibitory firing delay in these neurons (Liss et al., 2001; Putzier et al., 2009b; Amendola et al., 2012; Tarfa et al., 2017). In addition, single-cell PCR, in situ hybridization and immunohistochemistry experiments suggested that the A-type current is carried exclusively by Kv4.3 ion channels (Serôdio and Rudy, 1998; Liss et al., 2001; Ding et al., 2011; Dufour et al., 2014a; Tapia et al., 2018). Interestingly, several studies also suggested that the H-type current (I_H_; carried by HCN channels) displays strong functional interactions with I_A_, having for instance an opposite influence on postinhibitory rebound delay (Amendola et al., 2012; Tarfa et al., 2017). The gating properties of these two currents were also shown to be co-regulated in rat SNc DA neurons (Amendola et al., 2012).

In the current study, we used in particular electrophysiological recordings from wild-type (WT) and Kv4.3−/− mice to refine the identity and role of Kv4 channels in the firing of SNc DA neurons. Using this mouse model, we show that the constitutive loss of Kv4.3 is associated with increased exploratory activity and impaired motor learning. Consistently, it is also associated with a lack of compensatory changes in other ion currents at the cellular level. We then demonstrate that the Kv4.2 subunit is expressed in SNc DA neurons, although its functional contribution is minor in most SNc DA neurons. Finally, we also demonstrate that pacemaking frequency and postinhibitory rebound delay are mainly determined by I_A_ time constant of inactivation and I_H_ amplitude.

## Materials and Methods

### Animals

Female and male postnatal day (P)15–P80 WT (*n* = 68 animals) and Kv4.3−/− (*n* = 40, Deltagen) mice from C57BL6/J genetic background were housed with free access to food and water in a temperature-controlled room (24°C) on a 12/12 h light/dark cycle (lights on at 7 A.M.). All efforts were made to minimize the number of animals used and to maintain them in good general health, according to the European (Council Directive 86/609/EEC) and institutional guidelines for the care and use of laboratory animals (French National Research Council).

### Behavioral experiments

A group of female and male WT (*n* = 11) and Kv4.3−/− (*n* = 13) mice aged P56-P63 at the start of the behavioral testing were used to evaluate changes in motor function, using in particular locomotor activity chambers and the rotarod test.

#### Locomotor and exploratory activities

Actimetry was monitored in individual activity chambers (20 × 11.2 × 20.7 cm) housed within a sound-attenuating cubicle and under homogeneous illumination (Imetronic). Each chamber was equipped with two pairs of infrared photobeams located 1.5 and 7.5 cm above the floor level of the chamber. The number of back-and-forth movements (animals breaking the lower photobeams) as well as the number of vertical movements (animals breaking the upper photobeams) were recorded in 5-min bins over 90 min. Numbers of back-and-forth movements (locomotion) and vertical movements (rearing) are shown as mean ± SEM for each time bin over the whole period of recording time. 

#### Motor learning

Motor learning was evaluated on the accelerating rotarod (10-cm diameter rod) test at a speed of 5–40 rotations per min (RPM) for 5 min. On the first day, mice were allowed to freely explore the non-rotating apparatus for 60 s and subsequently trained to hold on the rotating rod (5 RPM) for at least two 60-s trials, each trial being separated by a 10-min break. Mice were allowed to recover for 1 h before the first test. The testing phase consisted in 10 consecutive trials on the accelerating rod separated by 15-min breaks that allowed consolidation of performance. Results are shown as the average latency to fall off the rod (mean ± SEM) at each trial. A performance index was calculated for each individual and consisted in the average latency of the last three trials divided by the average latency of the first three trials multiplied by 100 ($\frac{averagelatency8-10}{averagelatency1-3}\times 100$).

### Electrophysiology

A total of 119 neurons from 39 WT mice and 109 neurons from 24 Kv4.3−/− mice were recorded (current-clamp and voltage-clamp).

#### Acute midbrain slice preparation

Acute slices were prepared from P15 to P25 animals of either sex. Mice were anesthetized with isoflurane (CSP) in an oxygenated chamber (TEM SEGA) before decapitation. After decapitation the brain was immersed briefly in oxygenated ice-cold low-calcium artificial CSF (aCSF) containing the following: 125 mm NaCl, 25 mm NaHCO_3_, 2.5 mm KCl, 1.25 mm NaH_2_PO_4_, 0.5 mm CaCl_2_, 4 mm MgCl_2_, and 25 mm D-glucose, pH 7.4, oxygenated with 95% O_2_/5% CO_2_ gas. The cortices were removed and then coronal midbrain slices (250 μm) were cut in ice-cold oxygenated low-calcium aCSF on a vibratome (Leica VT1200S and vibrating microtome 7000smz, Camden Instruments). Following 20- to 30-min incubation in oxygenated low-calcium aCSF at 33°C, the acute slices were then incubated for a minimum of 30 min in oxygenated aCSF (containing the following: 125 mm NaCl, 25 mm NaHCO_3_, 2.5 mm KCl, 1.25 mm NaH_2_PO_4_, 2 mm CaCl_2_, 2 mm MgCl_2_, and 25 mm glucose, pH 7.4, oxygenated with 95% O_2_/5% CO_2_ gas) at room temperature (RT) before electrophysiological recordings.

#### Drugs

Kynurenate (2 mm, Sigma-Aldrich) and picrotoxin (100 μm, Sigma-Aldrich) were used to block excitatory and inhibitory synaptic activity, respectively. AmmTX3 (1 μm, Alomone) was used to block the transient potassium current (I_A_) carried by Kv4 channels. Drugs were bath applied via continuous perfusion in aCSF.

#### Electrophysiology recordings and analysis

All recordings (228 neurons from 63 mice) were performed on midbrain slices continuously superfused with oxygenated aCSF at 30–32°C. Picrotoxin and kynurenate were systematically added to the aCSF for all recordings to prevent contamination of the intrinsically generated activity by glutamatergic and GABAergic spontaneous synaptic activity. Patch pipettes (1.9–2.7 MΩ) were pulled from borosilicate glass (GC150TF-10, Harvard Apparatus) on a DMZ-Universal Puller (Zeitz Instruments) and filled with a patch solution containing the following: 20 mm KCl, 10 mm HEPES, 0.5 mm EGTA, 2 mm MgCl_2_, 0.4 mm Na-GTP, 2 mm Na_2_-ATP, 4  mm Mg-ATP, 0.3 mm CaCl_2_, SUPERase RNase inhibitor (0.1 U/μl), and 115 mm K-gluconate, pH 7.4, 290–300 mOsm. For AmmTX3 experiments, patch pipettes (3.2–4.0 MΩ) were filled with a patch solution containing the following: 20 mm KCl, 10 mm HEPES, 0.5 mm EGTA, 2 mm MgCl_2_, 2 mm Na-ATP, and 120 mm K-gluconate, pH 7.4, 290–300 mOsm. Whole-cell recordings were made from SNc DA neurons visualized using infrared differential interference contrast videomicroscopy (QImaging Retiga camera; Olympus BX51WI microscope), and were identified based on their location, large soma size (>25μm), and electrophysiological profile (regular slow pacemaking activity, large spike half-width, large sag in response to hyperpolarizing current steps). For voltage-clamp experiments, only whole-cell recordings with an uncompensated series resistance <7 MΩ (compensated 85–90%) were included in the analysis. For current-clamp pharmacology experiments, higher series resistances were tolerated as long as the bridge compensation was properly adjusted to 100%. Liquid junction potential (−13.2 mV) and capacitive currents were compensated on-line. Recordings were acquired at 50 kHz and were filtered with a low-pass filter (Bessel characteristic 2.8-kHz cutoff). For current-clamp recordings, 1-s hyperpolarizing current steps were injected to elicit a hyperpolarization-induced sag (because of I_H_ activation).

#### Current-clamp recordings and protocols

The spontaneous firing frequency was calculated from a minimum of 30 s of stable recording in cell-attached mode and from current-clamp recording (with no injected current) within the first 5 min after obtaining the whole-cell configuration. The coefficient of variation of the interspike interval (CV_ISI_) was extracted from the same recording. APs generated during this period of spontaneous activity were averaged and several parameters were extracted: AP threshold, AP amplitude, AP duration at half of its maximal height (AP half-width), afterhyperpolarization (AHP) trough voltage, AHP latency. Hyperpolarizing current steps and depolarizing current steps were used to characterize the postinhibitory rebound and the excitability properties. The gain start, gain end and spike frequency adaptation (SFA) index used to define excitability were calculated as described before (Dufour et al., 2014b).

#### Voltage-clamp recordings

The I_A_ current was elicited by a protocol consisting in a 500-ms prestep at −100 mV (to fully de-inactivate I_A_) followed by a 500-ms voltage step to −40 mV (to activate I_A_ without eliciting delayed rectifier potassium currents). The current generated by the same protocol using a prestep at –40 mV (to fully inactivate I_A_) was subtracted to isolate I_A_. I_A_ properties (peak amplitude and total charge) were measured after subtracting the baseline at –40 mV. Total charge was calculated by integrating the current over the whole duration (500 ms) of the voltage step. The peak of the current elicited at –40 mV was then plotted against the voltage of each corresponding prestep, and was fitted with a Boltzmann function to obtain I_A_ half-inactivation voltage (V_50_ I_A_; see Amendola et al., 2012). The inactivation time constant (I_A_ tau) was extracted from a mono-exponential fit of the decay of the current. A two-step voltage-clamp protocol was used to determine the voltage dependence of activation of I_H_ (V_50_ I_H_) and obtain the maximum I_H_ amplitude (for details, see Amendola et al., 2012). For voltage-clamp recordings of delayed rectifier current (I_KDR_), tetrodotoxin (1 μm, Alomone), nickel (200 μm, Sigma-Aldrich), and cadmium (400 μm, Sigma-Aldrich) were also added to the aCSF. I_KDR_ was elicited by using a protocol consisting in a prestep at –40 mV (to fully inactivate I_A_) followed by incremental depolarizing voltage steps up to +40 mV.

#### Data acquisition

Data were acquired using an EPC 10 USB patch-clamp amplifier (HEKA) and the Patchmaster software acquisition interface (HEKA). Analysis was performed using FitMaster v2x73 (Heka).

### Immunohistochemistry

Adult (P21–P28) C57BL/6 WT mice (*n* = 2) or Kv4.3−/− littermates (*n* = 2) of either sex were euthanized with ketamine-xylazine mix (100 mg/kg ketamine, 10 mg/kg xylazine), and transcardially perfused with PBS and ice-cold 4% paraformaldehyde in PBS. Brains were removed and postfixed overnight (o/n) at 4°C in the same fixative solution. Coronal brain slices of 50 μm were obtained using a vibratome (vibrating microtome 7000smz, Camden Instruments) and collected as floating sections. When indicated, antigen retrieval (AR) was performed by incubating the slices in sodium citrate (10 mm, Sigma-Aldrich) during 30 min at 80°C (Jiao et al., 1999). Subsequently, slices were blocked for 1 h 30 min at RT in a solution containing 0.3% Triton X-100 (Sigma) and 5% normal goat serum (NGS; Vector Laboratories) in PBS. After blocking, sections were incubated with primary antibodies in a solution containing 0.3% Triton X-100 and 1% NGS in PBS (o/n; 4°C). The following primary antibodies were used in this study: chicken anti-TH (1:1000; Abcam, ab76442, RRID:AB_1524535), rabbit anti Kv4.3 (1:500, Alomone Labs, APC-017, RRID:AB_2040178), and mouse anti Kv4.2 (1:200, Neuromab, 75-361, clone L28/4, RRID:AB_2315873). After three washes (15 min/each) in PBS containing 0.3% Triton X-100, the floating sections were incubated with the following secondary antibodies: Alexa Fluor 488-goat anti-mouse (1:200, Jackson ImmunoResearch), Alexa Fluor 488-goat anti-rabbit (1:200, Jackson ImmunoResearch) and Alexa Fluor 594-goat anti-chicken (1:200, Jackson ImmunoResearch) in a PBS solution containing 0.3% Triton X-100 and 1% NGS for 2 h at RT. Finally, sections were washed three times (15 min/each), incubated with DAPI (1.5 μg/ml; Sigma-Aldrich) for 10 min, and mounted in Vectashield (Vector Laboratories). Sections were stored at 4°C, and images were acquired on a Zeiss LSM-780 confocal scanning microscope. All experiments involving WT and Kv4.3−/− comparisons were performed in parallel applying the same acquisition settings to both genotypes. Images were processed and analyzed with ImageJ (NIH). Kv4.2-positive cells were visually identified in both genotypes on the basis of a perimembranous-like Kv4.2 staining and expressed as a percentage of the total number of TH+ cells. In order to compare the labeling pattern of Kv4.2 and Kv4.3, the line selection tool was used to trace 3-μm-length lines perpendicular to the cell perimeter in individual optical sections. In each cell, three regions were analyzed, and five cells were used to calculate the average profile in each condition. Raw intensity values were collected, normalized (0–1 range) to the maximal value, and plotted as a function of distance (0 corresponding to Kv4 peak fluorescence signal, negative distances to extracellular space and positive distances to intracellular space). All the images shown are one single optical slice. 

### Modeling

Simulations were performed using NEURON 7.5 software (Hines and Carnevale, 2001) as previously described (Moubarak et al., 2019). Realistic morphologies of 22 rat SNc DA neurons obtained previously were used to build multicompartment models (Moubarak et al., 2019). For each compartment, membrane voltage was obtained as the time integral of a first-order differential equation:

$$\frac{dV}{dt}=-\frac{1}{C_{m}}*\sum^{}\left[g_{i}*\left(V_{m}-E_{rev}\right)\right]-I_{axial},$$

where V_m_ is the membrane potential, C_m_ the membrane capacitance, g_i_ are ionic conductances and E_rev_ their respective reversal potentials. The axial flow of current (I_axial_) between adjacent compartments is calculated by the NEURON simulation package (Hines and Carnevale, 2001). Cytoplasmic resistivity, specific membrane capacitance and specific membrane resistance were set to 150 Ω/cm, 0.75 μF/cm^2^, and 100,000 Ω/cm^2^, respectively, with E_rev_ for the leak conductance set at −50 mV. Six active conductances were included in the model: fast sodium (I_Na_), delayed rectifier potassium (I_KDR_), transient potassium (I_A_), L-type calcium (I_CaL_), hyperpolarization-activated (I_H_), and small conductance calcium-activated potassium (I_SK_) currents (Moubarak et al., 2019). Active conductances followed activation-inactivation Hodgkin–Huxley kinetics (Table 1). Parameters for I_A_, I_CaL_, I_SK_, I_Na_, I_KDR_, and I_H_ were based on our previous model and published values for SNc DA neurons (Gentet and Williams, 2007; Seutin and Engel, 2010; Amendola et al., 2012; Philippart et al., 2016; Moubarak et al., 2019). Intracellular calcium uptake was modeled as a simple decaying model according to Destexhe et al. (1993). Conductance values were set according to our own measurements or published values (see Table 1). Consistent with the literature (Kole and Stuart, 2008; Hu et al., 2009), g_Na_ and g_KDR_ were set to higher values in the axon initial segment (AIS) than in the rest of the neuron so that the AP always initiated in the AIS. For sake of simplicity, activation and inactivation kinetics of I_A_ were voltage-independent but coupled to each other, such that activation rate was 50 times faster than inactivation rate. In addition, the inactivation and activation V_50_s were also coupled (50-mV shift). As I_A_ and I_H_ voltage dependences have been shown to be positively correlated in rat SNc DA neurons (Amendola et al., 2012), both values were forced to co-vary in the model according to the equation V_50_ inact. I_A_ = 0.814 × (V_50_ act. I_H_) + 3.36.

**Table 1:** Equations governing the voltage dependence and kinetics of currents in the model

Current general equations (except for I_SK_)		
$I\left(V,t\right)=g_{max}\times m^{a}\left(V,t\right)\times h^{b}\left(V,t\right)\times \left(V-E_{rev}\right)$		
$m_{\infty }\left(V\right)=\frac{1}{\left(1+exp\left[\left(\frac{-\left(V-V_{m}\right)}{k_{m}}\right)\right]\right)}$ $dm\left(V,t\right)/dt=\frac{\left[m_{\infty }\left(V\right)-m\left(V,t\right)\right]}{\tau_{m}}$					$h_{\infty }\left(V\right)=\frac{1}{\left(1+exp\left[\left(\frac{-\left(V-V_{h}\right)}{k_{h}}\right)\right]\right)}$ $dh\left(V,t\right)/dt=\frac{\left[h_{\infty }\left(V\right)-h\left(V,t\right)\right]}{\tau_{h}}$					*dt* = 20 μs	
Current	V_m_ (mV)	k_m_ (mV)	a	V_h_ (mV)	k_h_ (mV)	b	E_rev_ (mV)	Specific equations (τ_m_ and τ_h_ are expressed in ms)	g_max_(pS/μm^2^)		
									Soma and dendrites	AIS	Axon
I_Na_	–28	8	3	–50	–10	1	60	τ_m_ = 0.01+(0.33/(1+((V+20)/30)^2^)) τ_h_ = 0.7+(16/(1+((V+50)/8)^2^))	75	4000	400
I_KDR_	–30	9	4	-	-	0	–90	τ_m_ = (4* exp(-(0.000729)*((V +32)^2^))) + 4	150	4000	400
I_A_	V_h_+50	7	1	–78 to –62	–7	1	–90	τ_m_ = τ_h_/50 τ_h_ = 15-150	15–150	0	0
I_H_	–100 to –80	–7.25	1	-	-	0	–40	τ_m_ = 556+ 1100* exp (-0.5*((V)/11.06)^2^)	0.25–2.5	0	0
I_CaL_	–31	7	1	-	-	0	120	τ_m_ = 1/((-0.209*(v+39.26) /(exp(-(v+39.26)/4.111)-1)+ (0.944*exp(-(v+15.38)/224.1))))	1	0	0
I_SK_	-	-	-	-	-	-	–90	$I\left(V,Ca_{i}\right)=g_{max}\times O_{\infty }\left(Ca_{i}\right)\times \left(V-E_{rev}\right)$ O_∞_(V) = (Ca_i_)^4^ /((Ca_i_)^4^ + (0.00019)^4^)	0.125	0	0
**Calcium buffering and pump** (see Desthexe et al., 1993)											

Initializing potential was set at −70 mV and pacemaking frequency was let to stabilize (four spikes) before further analysis. Each simulation run had a duration of 8000 ms with a dt of 0.02 ms. Spatial discretization followed the “d_lambda rule” (Hines and Carnevale, 2001). All dendritic compartments and the axon-start compartment contained all currents whereas AIS and axon only contained fast sodium (I_Na_) and delayed rectifier potassium (I_KDR_) currents. To measure postinhibitory rebound delay, current injection was performed by inserting a virtual electrode in the soma. A 1-s pulse of current was injected into the model. Negative current amplitude was adjusted to achieve a peak hyperpolarization around −120 mV in each neuron and condition. Firing frequency, rebound delay and AP property analyses were computed online by handmade routines directly written in NEURON hoc language (Hines and Carnevale, 2001). This model is derived from a previous model available at model DB database under the number 245427.

### Statistics

All statistical analyses were conducted under the R environment with appropriate packages. For behavioral experiments, normality was assessed by visual inspection of quantile-quantile (Q-Q) plots for the different scores per animal (ggpubr package). Nearly all the data points did not depart for normality estimated within a 95% coefficient interval. For electrophysiology experiments, normality was checked using the Shapiro–Wilk normality test (stat package).

#### Behavior

To assess locomotor and exploratory behavior, number of horizontal (locomotion) and vertical (rearing) photobeam breaks was measured per 5-min bin over 90 min and compared between genotypes. As no significant difference was found between males and females in any of the behavioral tests, both sexes were pooled and analyzed as a single sample. Data are represented as line and scatter plots for the number of horizontal and vertical photobeam breaks per 5-min bin. To assess the locomotor phenotype of Kv4.3−/− mice, two-way repeated measures ANOVA tests with groups (WT/Kv4.3−/−) as the independent between-factor and time as the within-factor (training sessions or time-bins for locomotion) were performed (stat package). When the ANOVA was significant, multiple comparisons [false-discovery rate (fdr) adjustment, multicomp and emmeans packages] were used to evaluate differences between groups at different time points (Benjamini and Hochberg, 1995); *p* < 0.05 was considered as statistically significant for all analyses. For motor learning, the average latency to fall off the accelerating rotarod was measured for each trial. Statistical difference in motor learning was assessed by comparing the performance index. Data are represented as line and scatter plots for the average latency to fall off the rod. Data are represented as mean ± SEM.

#### Electrophysiology and immunohistochemistry

The univariate statistical analysis of electrophysiological data, performed according to the distribution properties of the data using a Shapiro–Wilk normality test, included paired *t* test or Wilcoxon signed-rank test; *t* test or Mann–Whitney Wilcoxon test with *p* < 0.05 considered to be statistically significant (stat package). In most figures, data are represented as scatter plots or box and whisker plots, with all individual points appearing on the graphs and dotted lines indicating the distribution of data (violin plots). For pharmacological experiments, data are represented as mean ± SEM (scatter or bar plots). Correlation, linear regression and multiple linear regression analysis were performed in *R*. For every pair of variables, correlation parameters, ρ (Spearman correlation factor) or *r* (Pearson correlation factor), were selected after performing a Shapiro–Wilk normality test on the linear regression residuals and *p* values were corrected for multiple comparisons by an fdr adjustment (stat package). For multiple linear regression, variables (extracellular ISI, rebound delay, I_A_ tau, I_H_ amplitude, and I_A_ amplitude) were first log transformed, and then dependent variables were standardized (subtracting the mean and dividing by the SD). A selection of the best subsets of dependent variables for each model size (1–4 for the model and 1–5 for real data) was first performed (leaps package) according to several criteria (adjusted *R*^2^, AIC, BIC). The best model was then selected by comparing the prediction error of each model after performing a repeated (20 times) 10-fold cross-validation on test data (caret package). The best linear model, corresponding to the minimum cross-validation error (i.e., a model with the best predictive power) was then obtained. Multicolinearity was assessed by computing a score called the variance inflation factor (VIF package) and VIF was <1.5 for all variables retained in the different models. For the immunohistochemistry experiments, a Fisher’s exact test was used to compare the proportions of Kv4.2-positive cells among TH-positive cells in WT and Kv4.3−/− mice. Figures were prepared using R, SigmaPlot 11.0, GraphPad Prism 6, and Adobe Illustrator CS5/CS6.

**Figure 1.**
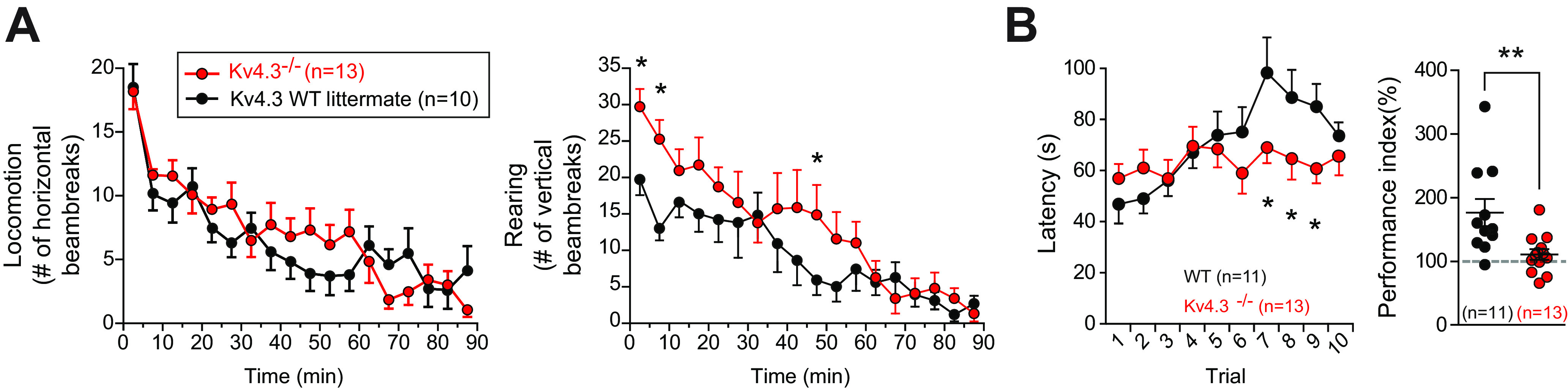
Behavioral assessment of the Kv4.3−/− transgenic mouse. ***A***, Locomotion measured in actimetry chambers. Left, Line and scatter plot showing the mean number of horizontal movements per 5-min bin (±SEM) in Kv4.3−/− mice (red) compared with their WT littermates (black). Right, Line and scatter plot showing the mean number of rearing events per 5-min bin in Kv4.3−/− mice (red) compared with their WT littermates (black). Significant differences between strains for specific time bins (two-way repeated measures ANOVA) are indicated by an asterisk. ***B***, Changes in motor learning measured using a rotarod assay. The latency to falling off the rotating rod (with increasing rotating speed) was assessed over 10 consecutive trials (left). Significant differences between strains for specific trials (two-way repeated measures ANOVA) are indicated by an asterisk. The performance index [(average latency 8–10)/(average latency 1–3) × 100; right] was used to evaluate the learning ability of Kv4.3−/− mice (red) compared with their WT littermates (black); *p < 0.05, **p < 0.01.

## Results

### Motor learning impairment of the Kv4.3−/− transgenic mice

In order to define the precise contribution of Kv4.3 channels to SNc DA neuron electrophysiological phenotype, we used Kv4.3 constitutive knock-out (KO) mice (Niwa et al., 2008; Carrasquillo et al., 2012; Granados-Fuentes et al., 2012). These mice have been used previously to study Kv4.3 function in cardiac ventricles (Niwa et al., 2008), in neurons of the suprachiasmatic nucleus (Granados-Fuentes et al., 2012) and in neocortical pyramidal neurons (Carrasquillo et al., 2012). No major defect in cardiac function or circadian locomotor behavior was reported in these studies. As Kv4.3 is also strongly expressed in the SNc and in the ventral tegmental area (VTA; Serôdio and Rudy, 1998), we first sought to determine whether Kv4.3 loss could affect SNc- or VTA-related behaviors, such as locomotion and motor learning (Fig. 1). As illustrated in Figure 1*A*, horizontal locomotor activity assessed in photocell activity chambers was similar between Kv4.3−/− mice and their WT littermates (*F*_(1,21)_ = 0.31, *p* = 0.58, between factor effect), both strains displaying a significant decrease of locomotion over time (*F*_(17,357)_ = 20.5, *p* = 2 × 10^−16^, within factor effect). The exploratory behavior was higher in Kv4.3−/− mice than WT littermates in particular during the first part of the session (Fig. 1*A*), although the overall exploratory activity was not significantly different between the two strains (*F*_(1,21)_ = 1.42, *p* = 0.25, between factor effect). Again, both strains displayed a strong run-down of activity over the whole 90-min testing time as a function of habituation to the locomotor activity chambers (*F*_(17,357)_ = 13.97, *p* = 2 × 10^−16^, within factor effect). Consistent with the increased exploratory activity at the start of the session for the Kv4.3−/− mice, the two-way repeated measures ANOVA revealed a significant interaction between strains and time (*F*_(17,357)_ = 1.87, *p* = 0.019). Motor learning abilities were then assessed using the rotarod test for 10 consecutive trials (Fig. 1*B*). While WT mice improved their performance across trials, as shown by the increase in the latency-to-fall, Kv4.3−/− mice were not able to adjust their performance over time. While the latency averaged over trials was not different between the two strains (*F*_(1,22)_ = 1.26, *p* = 0.28, between factor effect, two-way repeated measures ANOVA), performance changed significantly across trials (*F*_(9,198)_ = 4.5, *p* = 2 × 10^−5^, within factor effect), especially for WT mice. Consistent with the differences in latency for the late trials (Fig. 1*B*), the difference in learning between the two strains was revealed by the strains × trials interaction statistics (*F*_(9,198)_ = 2.95, *p* = 0.0026) and significant differences in fall-off latencies of Kv4.3−/− and WT mice was found for the late trials (7–9; Fig. 1*B*). In order to better quantify the difference in learning between strains, we then calculated a performance index based on the difference in latency for the first three trials against the last three trials. Consistent with the ANOVA results, the performance index in this motor learning task was significantly lower for the Kv4.3−/− mice (WT, 176.9 ± 21.4, *n* = 11 vs Kv4.3−/−, 111.2 ± 8.3, *n* = 13, *p* = 0.006, unpaired *t* test; Fig. 1*B*).

### Changes in electrophysiological phenotype of Kv4.3−/− SNc DA neurons

**Figure 2.**
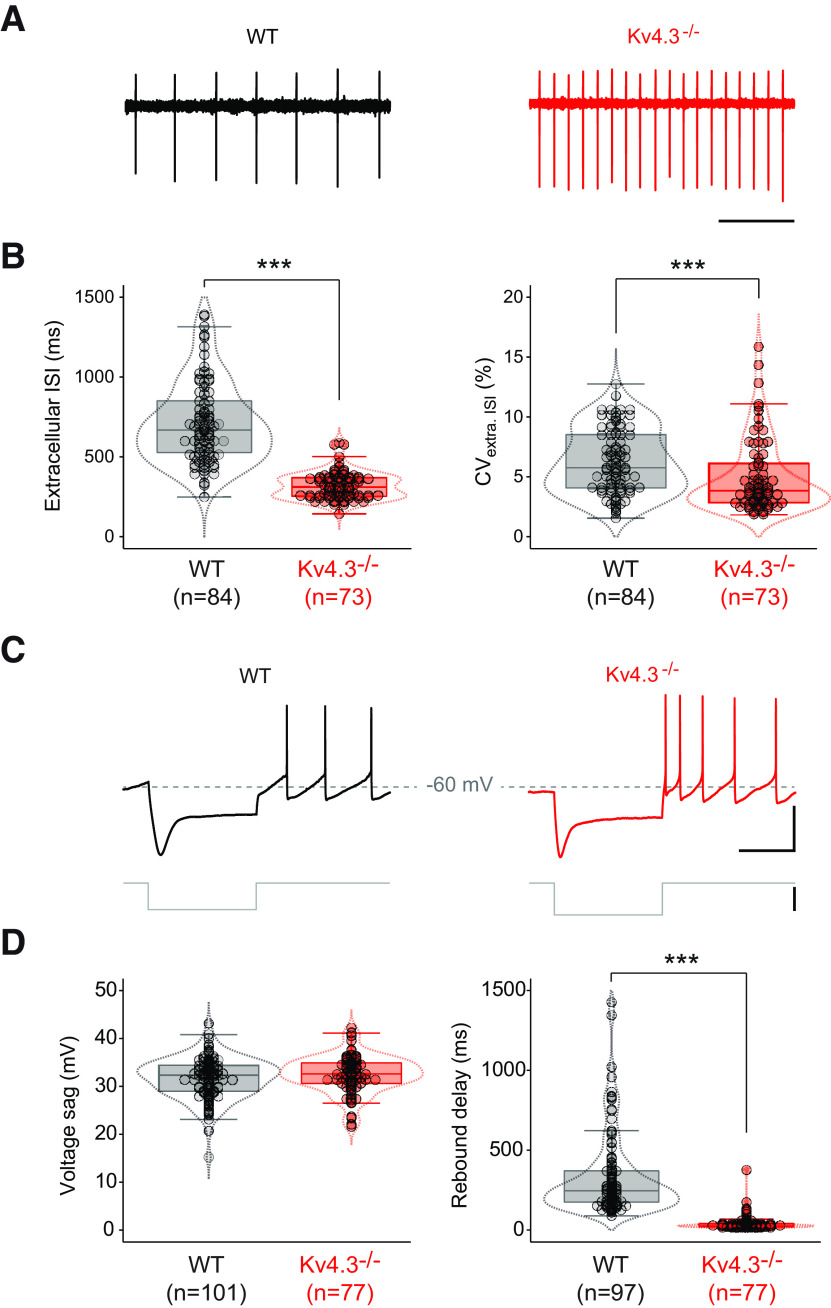
Spontaneous activity and postinhibitory rebound are profoundly altered in Kv4.3−/− SNc DA neurons. ***A***, Representative cell-attached recordings showing the spontaneous pattern of activity in WT (black trace, left) and Kv4.3−/− SNc DA neurons (red trace, right). ***B***, Box and whisker plots showing the distribution of values for extracellularly recorded spontaneous ISI (extracellular ISI; left) and ISI CV (CV_extra. ISI_; right) in WT and Kv4.3−/− SNc DA neurons. ***C***, Representative current-clamp recordings showing the voltage response of SNc DA neurons to a current step (gray trace) hyperpolarizing membrane voltage to ∼–120 mV in WT (black trace, left) and Kv4.3−/− mice (red trace, right). The recordings come from the same neurons as the cell-attached recordings presented in ***A***. ***D***, Box and whisker plots showing the distribution of values for voltage sag amplitude (left) and postinhibitory rebound delay (right) in WT and Kv4.3−/− SNc DA neurons; ***p < 0.001. Dotted lines in the box and whisker plots indicate the distribution of data (violin plots). Scale bars: 1 s (***A***, horizontal), 500 ms (***C***, top, horizontal), 40 mV (***C***, top, vertical), 100 pA (***C***, bottom, vertical), and –60 mV (***C***, horizontal dotted lines).

**Figure 3.**
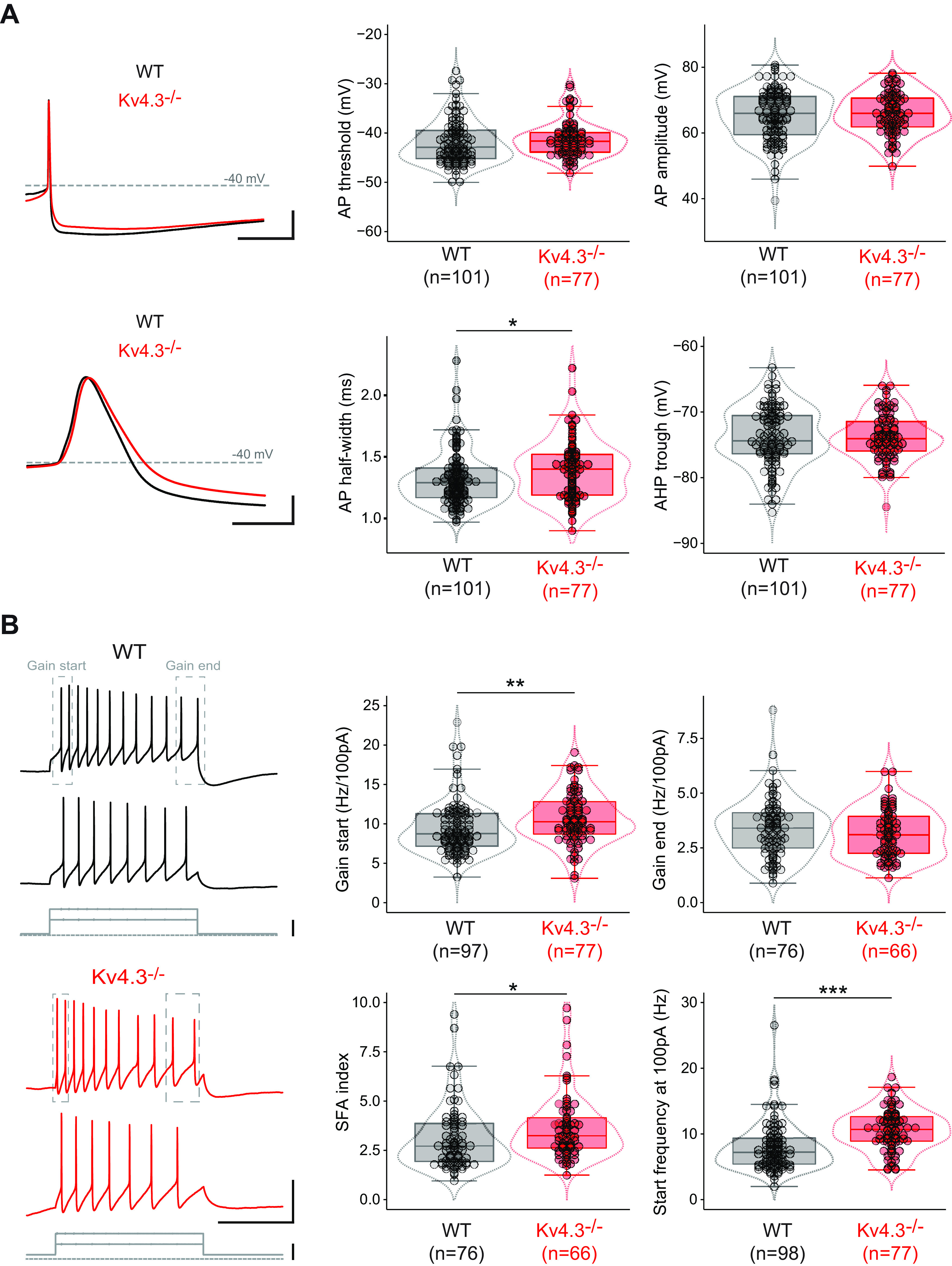
AP and excitability properties in Kv4.3−/− SNc DA neurons. ***A***, left, Current-clamp recordings showing the shape of the AP in the WT (black traces) and Kv4.3−/− mice (red traces) on a slow (top) and fast time-scale (bottom). Right, Box and whisker plots showing the distribution of values for AP threshold (top left), amplitude (top right), half-width (AP half-width, bottom left), and AHP trough (bottom right) in WT and Kv4.3−/− SNc DA neurons. ***B***, left, Current-clamp recordings showing the voltage response of SNc DA neurons to 100- and 200-pA depolarizing current steps (gray traces) in WT (top, black traces) and Kv4.3−/− mice (bottom, red traces). Gray dotted rectangles indicate the ISI used to calculate the gain start and gain end on each train of APs. Right, Box and whisker plots showing the distribution of values for gain start (top left), gain end (top right), SFA index (bottom left), and start frequency at 100 pA (bottom right) in WT and Kv4.3−/− SNc DA neurons; *p < 0.05, **p < 0.01, ***p < 0.001. Dotted lines in the box and whisker plots indicate the distribution of data (violin plots). Scale bars: 50 ms (***A***, top, horizontal), 20 mV (***A***, top, vertical), 2 ms (***A***, bottom, horizontal), 20 mV (***A***, bottom, vertical), –40 mV (***A***, horizontal dotted lines), 500 ms (***B***, voltage, horizontal), 40 mV (***B***, voltage, vertical), and 100 pA (***B***, current, vertical).

Following the approach already used in a previous study (Dufour et al., 2014b), we then performed an exhaustive current-clamp characterization of the firing properties of SNc DA neurons to determine changes in phenotype associated with Kv4.3 deletion (Table 2). Passive properties, spontaneous activity, postinhibitory rebound, AP shape and excitability were assessed by measuring 16 different electrophysiological parameters in 75–101 WT and 66–77 Kv4.3−/− neurons. The first obvious electrophysiological change observed was that spontaneous activity (extracellularly recorded in cell-attached mode) was dramatically modified in Kv4.3−/− SNc DA neurons (Fig. 2*A*,*B*). Spontaneous firing rate was increased by ∼2-fold in Kv4.3−/− mice, as demonstrated by the significant decrease in ISI (Fig. 2*A*,*B*; Table 2). Pacemaking regularity, measured by the CV_ISI_ was also significantly different in Kv4.3−/− mice, although CV_ISI_ values were very low (<20%) in both WT and Kv4.3−/− mice, indicating a highly regular tonic activity. Postinhibitory rebound delay was also dramatically decreased in Kv4.3−/− mice (Fig. 2*C*,*D*; Table 2). However, the I_H_-mediated voltage sag observed during prolonged hyperpolarization was not modified (Fig. 2*C*,*D*; Table 2). Interestingly, most AP parameters were unchanged in Kv4.3−/− mice, except for AP half-width, which was slightly larger (Fig. 3*A*; Table 2). We also analyzed neuronal excitability by measuring the responses of the neurons to increasing depolarizing current steps (Fig. 3*B*). Excitability was slightly increased in the DA neurons of Kv4.3−/− mice, although this change only affected the initial response of neurons (gain start) to current injection (Fig. 3*B*; Table 2). Consistently, the frequency of the response of Kv4.3−/− neurons to a 100-pA step was also found to be significantly higher (Fig. 3*B*; Table 2).

**Table 2.** Statistical analysis of electrophysiological parameters in wild-type and Kv4.3−/− **SNc DA neurons**

	WT			Kv4.3^–/–^			
Parameter	Mean/*median*	SD/*IQR*	*n*	Mean/*median*	SD/*IQR*	*n*	*p* value (*t* test) (*Mann–Whitney*)
**Passive properties**							
Rm (MΩ)	*386.00*	*202.00*	97	*422.00*	*147.00*	72	*0.2351*
**Pacemaking**							
ISI (ms)	*669.50*	*334.50*	84	*304.00*	*118.00*	73	2.2 × 10^–16^***
CV_ISI_ (%)	*5.76*	*4.45*	84	*3.84*	*3.31*	73	4 × 10^–4^***
**Postinhibitory rebound**							
Voltage sag (mV)	31.70	4.29	101	32.50	3.95	77	0.2082
Rebound delay (ms)	*256.00*	*199.00*	97	*29.00*	*18.00*	77	2.2 × 10^–16^***
**Action potential**							
AP threshold (mV)	*–42.90*	*5.80*	101	*–41.60*	*4.00*	77	*0.15*
AP amplitude (mV)	65.29	7.84	101	66.01	6.27	77	0.5024
AP half-width (ms)	*1.29*	*0.24*	101	*1.40*	*0.33*	77	0.038*
AP rise slope (mV/ms)	86.47	24.95	101	87.89	24.61	77	0.7049
AP decay slope (mV/ms)	–47.71	8.73	101	–45.33	8.89	77	0.0756
AHP trough V_m_ (mV)	–73.84	4.58	101	–73.83	3.58	77	0.9892
AHP latency (ms)	*55.56*	*38.28*	101	*53.76*	*28.68*	77	*0.84*
**Excitability**							
Start frequency 100 pA (Hz)	*7.23*	*3.98*	98	*10.68*	*3.73*	77	4 × 10^–8^***
Gain start (Hz/100 pA)	*8.75*	*4.18*	97	*10.27*	*4.11*	77	1.7 × 10^–3^**
Gain end (Hz/100 pA)	*3.41*	*1.61*	76	*3.11*	*1.69*	66	*0.37*
SFA index	*2.73*	*1.94*	76	*3.24*	*1.54*	66	0.012*
**I_A_**							
V_50_ inactivation (mV)	–68.91	5.10	87	–73.31	3.84	39	4.3 × 10^–6^***
Inactivation tau (ms)	*30.90*	*27.15*	83	*12.90*	*6.72*	42	7.1 × 10^–12^***
Amplitude (nA)	*2.14*	*2.79*	87	*0.93*	*0.64*	39	1.8 × 10^–12^***
Charge (pA.s)	*152.80*	*177.80*	83	*19.40*	*22.40*	37	6.4 × 10^–15^***
Charge density (pA.s/pF)	*1.17*	*1.00*	82	*0.18*	*0.20*	36	6.4 × 10^–13^***
**I_H_**							
V50 activation (mV)	–88.41	4.24	75	–88.42	3.81	55	0.9886
Amplitude (pA)	*453.00*	*212.50*	75	*575.30*	*275.80*	57	6 × 10^–4^***
Amplitude density (pA/pF)	*3.49*	*1.93*	74	*5.76*	*2.36*	54	7.6 × 10^–11^***

The values for 16 electrophysiological parameters measured under current-clamp (corresponding to passive properties, spontaneous activity, postinhibitory rebound, action potential, and excitability) and eight electrophysiological parameters measured under voltage-clamp (corresponding to I_A_ and I_H_ properties) are presented for WT and Kv4.3^−/−^ SNc DA neurons. Mean and SD (black text) are reported for normally-distributed data, while median and interquartile range (IQR) are reported otherwise (italic text). Accordingly, statistical differences between WT and Kv4.3^−/−^ neurons were tested using a *t* test or a Mann–Whitney test, depending on the normality of the data. Asterisks indicate statistically significant differences (*p < 0.05, **p < 0.01, ***p < 0.001).

### Voltage-clamp characterization of I_A_ in Kv4.3−/− SNc DA neurons

**Figure 4.**
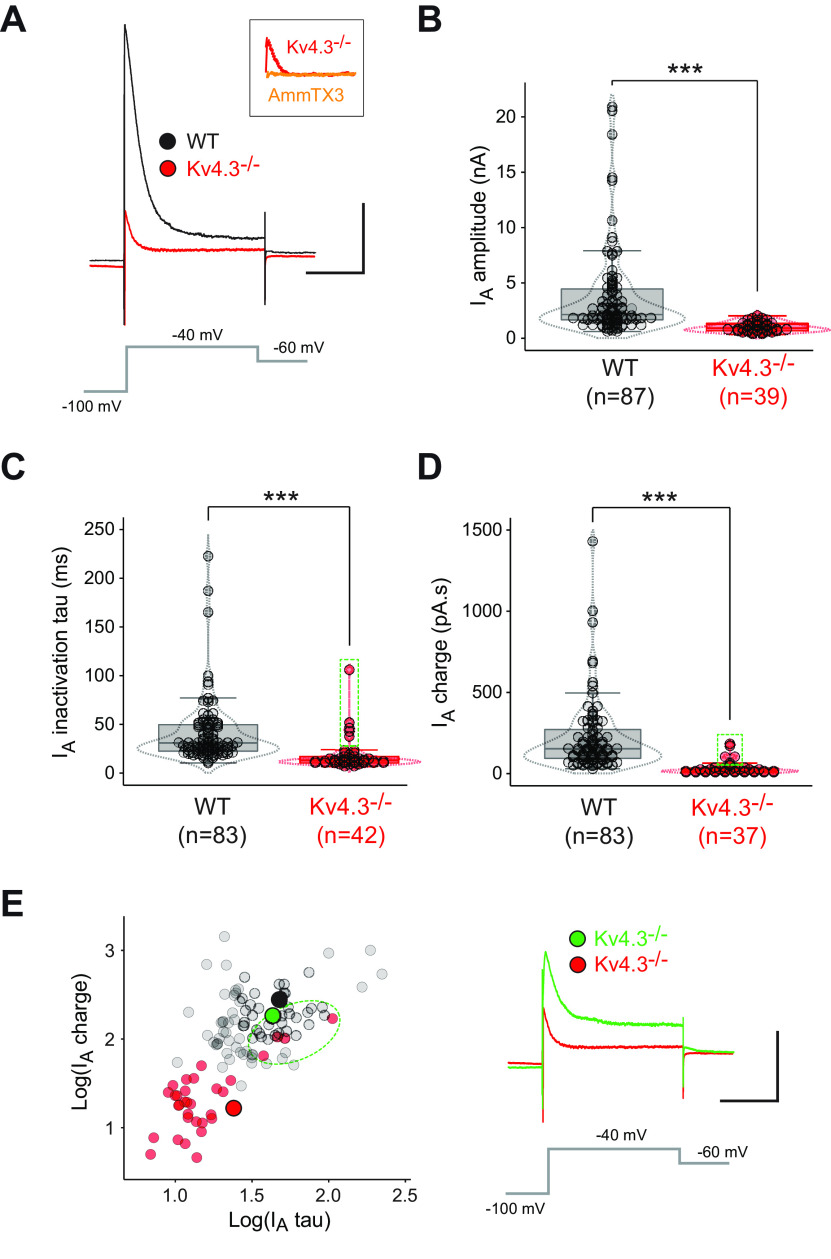
Voltage-clamp analysis of I_A_ in WT and Kv4.3−/− SNc DA neurons. ***A***, Voltage-clamp traces showing representative I_A_ recordings obtained from a WT (black trace) and a Kv4.3−/− SNc DA neuron (red trace) in response to a voltage step to –40 mV (gray trace). The small residual current present in the Kv4.3−/− mice is blocked by AmmTX3 (inset, orange trace). ***B***, Box and whisker plot showing the distribution of values for I_A_ amplitude in WT and Kv4.3−/− SNc DA neurons. ***C***, Box and whisker plot showing the distribution of values for I_A_ time constant of inactivation (I_A_ tau) in WT and Kv4.3−/− SNc DA neurons. The green dotted rectangle highlights five Kv4.3−/− outliers displaying unusually large values for I_A_ tau. ***D***, Box and whisker plot showing the distribution of values for I_A_ charge in WT and Kv4.3−/− SNc DA neurons. The green dotted rectangle highlights five Kv4.3−/− outliers displaying unusually large values for I_A_ charge (same cells as in ***C***). ***E***, left, Scatter plot showing the relationship between I_A_ tau and charge in WT (gray dots) and Kv4.3−/− SNc DA neurons (red dots). Please note that 5 of the Kv4.3−/− measurements lie in the WT region of space (green dotted ellipse). Right, Voltage-clamp traces showing one example of the atypical I_A_ recording (green trace, corresponding to the large green circle in the scatter plot) encountered in one of the 5 Kv4.3−/− outliers highlighted in panels ***C***, ***D***, compared with the typical recording obtained in Kv4.3−/− neurons (red trace, same as in panel ***A***, corresponding to the large red circle in the scatter plot); ***p < 0.001. Dotted lines in the box and whisker plots indicate the distribution of data (violin plots). Scale bars: 200 ms (***A***, ***E***, horizontal) and 1 nA (***A***, ***E***, vertical).

We then directly investigated changes in the properties of I_A_ by performing voltage-clamp recordings in WT and Kv4.3−/− SNc DA neurons (Fig. 4). A dramatic decrease in I_A_ amplitude was observed in Kv4.3−/− mice (Fig. 4*A*,*B*; Table 2). However, a small transient residual current with I_A_-like properties (voltage-dependent inactivation) was still present in all Kv4.3−/− recordings (Fig. 4*A*). Most interestingly, this residual current was completely blocked by the Kv4-specific toxin AmmTX3 (*n* = 4, no measurable residual current after toxin application; Fig. 4*A*), suggesting that a Kv4 subunit other than Kv4.3 is expressed at a low level in Kv4.3−/− SNc DA neurons. We then measured its time constant of inactivation (I_A_ tau) and calculated the overall charge carried by the current (Fig. 4*C–E*). Both parameters were strongly decreased in Kv4.3−/− SNc DA neurons (Fig. 4*C*,*D*; Table 2), although a minority of cells (*n* = 5/42) displayed values similar to the WT measurements for both of these parameters. Plotting I_A_ charge versus I_A_ tau revealed the clear separation of values between the Kv4.3−/− and WT measurements, except for the five cells identified before (Fig. 4*E*). Based on these voltage-clamp data, it appears that, although the Kv4.3 subunit by far predominates in WT SNc DA neurons, another Kv4 subunit is also expressed, at least in the Kv4.3−/− neurons. Although in most cases, the expression level of this unidentified subunit is too low to compensate for the loss of Kv4.3, it generates an A-type current that provides a minority of Kv4.3−/− SNc DA neurons (5/42 = 12%) with a “WT” voltage-clamp phenotype.

### Using AR to reveal the expression of Kv4.2 channels by SNc DA neurons

**Figure 5.**
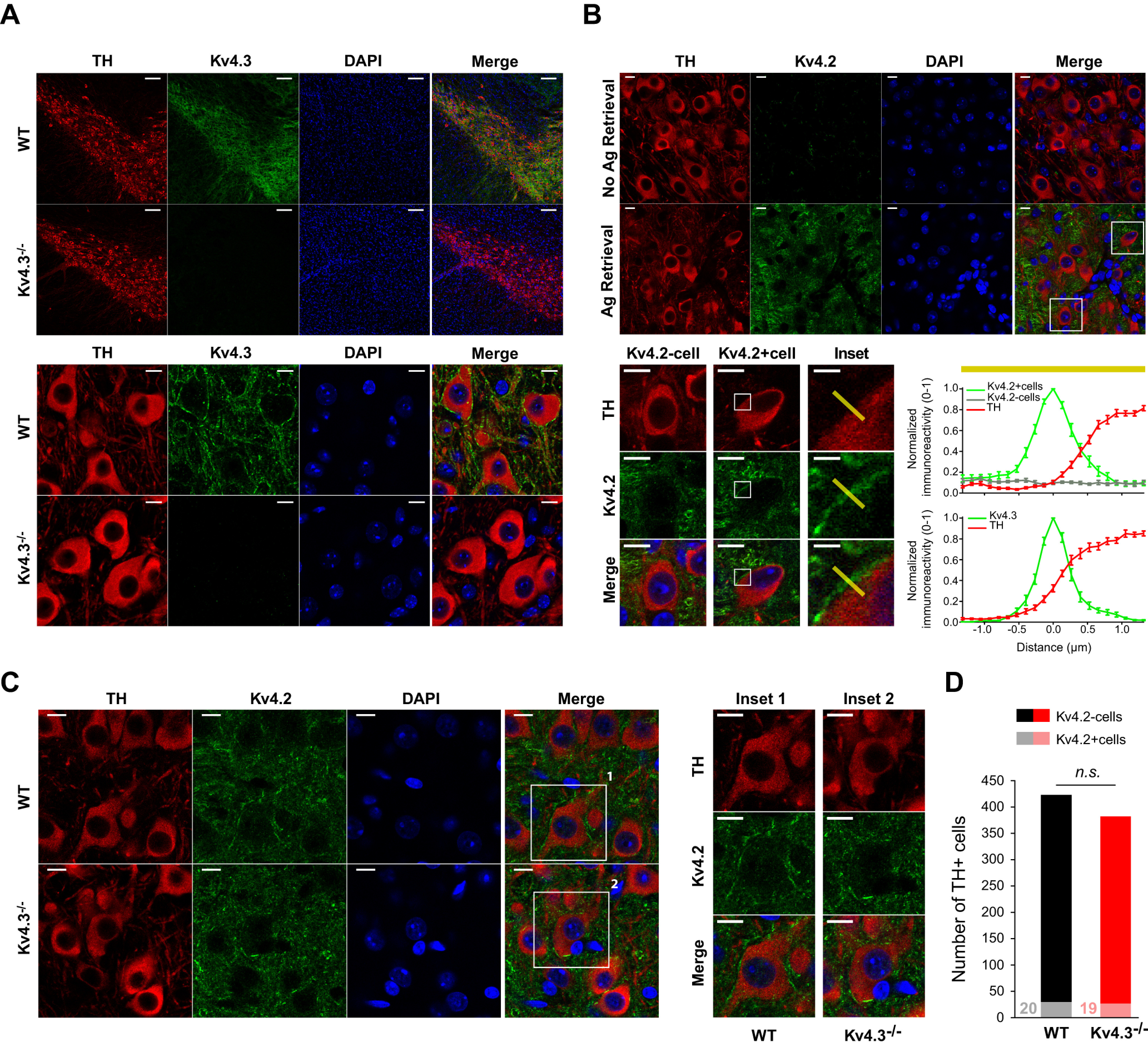
Kv4.2 channels are expressed by a minority of WT and Kv4.3−/− SNc DA neurons. ***A***, top, Low-magnification pictures of the SNc showing (from left to right) TH (red), Kv4.3 (green), and DAPI (blue) stainings in WT (top row) and Kv4.3−/− mice (bottom row). Bottom, High-magnification pictures of SNc DA neurons showing (from left to right) TH (red), Kv4.3 (green), and DAPI (blue) stainings in WT (top row) and Kv4.3−/− mice (bottom row). ***B***, Effect of the AR procedure on Kv4.2 detection in the SNc. Top, Low-magnification pictures showing (from left to right) TH (red), Kv4.2 (green), and DAPI staining (blue) without (top row) or with AR (bottom row). Bottom, left, High-magnification pictures of the insets depicted in the merged low-magnification picture and corresponding to a Kv4.2-negative (left) and a Kv4.2-positive SNc DA neuron (middle), for which inset pictures (right) illustrate the region selected to characterize the immunofluorescence profile of Kv4.2 and TH stainings (3 μm yellow bar). Bottom, right, Immunofluorescence profiles of Kv4.2 (green, top graph), Kv4.3 (green, bottom graph), and TH staining (red, both graphs) showing that both Kv4.2 and Kv4.3 profiles are very similar and strongly suggestive of specific plasma membrane expression. The average profiles (*n* = 5) shown here were defined over 3-μm selected regions of interest (yellow bar above the graph), such as the one shown on the inset pictures on the left. ***C***, left, High-magnification pictures showing (from left to right) TH (red), Kv4.2 (green), and DAPI staining (blue) after AR in WT (top row) and Kv4.3−/− mice (bottom row). Right, Expanded view of the Kv4.2-positive cells (1, 2) highlighted in the merged pictures on the left. To overcome differences regarding levels of TH expression in different DA neurons, different minimum and maximum display settings were applied for the TH channel. ***D***, Bar plot showing the counts of Kv4.2-negative (dark colors) and Kv4.2-positive (light colors) cells observed in the SNc of WT (black bar) and Kv4.3−/− mice (red bar). n.s., non-significant. Scale bars: 100 μm (***A***, top row), 10 μm (***A***, bottom row), 10 μm (***B***, top row), 10 μm (***B***, bottom row), 2 μm (***B***, inset), and 10 μm (***C***).

Several studies have nvestigated the expression of A-type Kv channels in SNc DA neurons, using in situ hybridization (Serôdio et al., 1996; Serôdio and Rudy, 1998), single-cell quantitative PCR (Liss et al., 2001; Ding et al., 2011; Tapia et al., 2018), and immunohistochemistry (Liss et al., 2001; Dufour et al., 2014a). A high level of expression for Kv4.3 (Liss et al., 2001; Dufour et al., 2014a; Tapia et al., 2018) and the absence of Kv4.1 (Serôdio and Rudy, 1998; Liss et al., 2001; Ding et al., 2011) were consistently reported, while the presence of Kv4.2 is still debated. In particular, while Kv4.2 mRNA has been detected in several studies (Ding et al., 2011; Tapia et al., 2018), the protein was not detected by classical immunohistochemistry (Liss et al., 2001; Dufour et al., 2014a). Interestingly, it has been shown that, depending on the brain region and the subcellular location of the ion channel of interest, an AR procedure may be required to uncover potassium channel antigen epitopes before performing immunolabeling (Lorincz and Nusser, 2008). We first confirmed that Kv4.3 was strongly expressed in SNc DA neurons in WT mice, with the expected membrane profile of immunostaining, and that it was absent from Kv4.3−/− SNc DA neurons (Fig. 5*A*). We then performed Kv4.2 immunolabeling with or without AR on the neocortex and the CA1 region of the hippocampus where this ion channel is highly expressed (Serôdio and Rudy, 1998). As can be seen in Extended Data Figure 5-1, Kv4.2 immunostaining was greatly improved by AR, revealing a strong perisomatic and dendritic staining of pyramidal cells in both regions. Therefore, we implemented AR before performing Kv4.2 immunostaining on WT midbrain slices (Fig. 5*B*). Similar to what we observed in the hippocampus and in the cortex, Kv4.2 immunostaining in the SNc was greatly improved by AR, although only a minority of DA neurons (TH-positive) displayed a clear perisomatic Kv4.2 signal compatible with membrane expression of the channel. In fact, the AR Kv4.2 staining profile of Kv4.2-positive cells was very similar to the membrane staining profile observed for Kv4.3 (Fig. 5*B*). This distinctive staining profile was then used to quantify the percentage of Kv4.2-positive SNc DA neurons in both WT and Kv4.3−/− mice (Fig. 5*C*,*D*). The percentage of Kv4.2-positive cells was not significantly different between WT and Kv4.3−/− mice (WT 20/423 = 4.7% vs Kv4.3−/− 19/382 = 5%, *p* = 1, Fisher’s exact test; Fig. 5*D*), suggesting that Kv4.2 pattern of expression is not modified by the loss of Kv4.3. This percentage is not statistically different from the percentage of Kv4.3−/− SNc DA neurons presenting an atypically large and slow I_A_ reported in Figure 4*C–E* (5/42 = 11.9% vs 19/382 = 4.6%, *p* = 0.077, Fisher’s exact test). Moreover, the percentage of Kv4.2-positive DA neurons was very similar in the medial and lateral SNc of both WT (SNc medial 11/227 = 4.8% vs SNc lateral 9/196 = 4.6%, *p* = 1, Fisher’s exact test) and Kv4.3−/− mice (SNc medial 13/218 = 6% vs SNc lateral 6/164 = 3.7%, *p* = 0.35, Fisher’s exact test).

10.1523/ENEURO.0207-21.2021.f5-1 Extended Data Figure 5-1 Validation of AR Kv4.2 immunohistochemistry on neocortical and hippocampal neurons. ***A***, Low-magnification (top rows) and high-magnification pictures (bottom rows) showing the effect of the AR procedure on Kv4.2 immunostaining (green) in the cortex. DAPI staining (blue) is also shown. **B**, Low-magnification (top rows) and high-magnification pictures (bottom rows) showing the effect of the AR procedure on Kv4.2 immunostaining (green) in the CA1 region of the hippocampus. DAPI staining (blue) is also shown. Scale bars: 100 μm (**A**, top row), 10 μm (***A***, bottom row), 100 μm (***B***, top row), and 10 μm (***B***, bottom row). Download Figure 5-1, EPS file.

### Lack of compensation in the face of Kv4.3 loss

**Figure 6.**
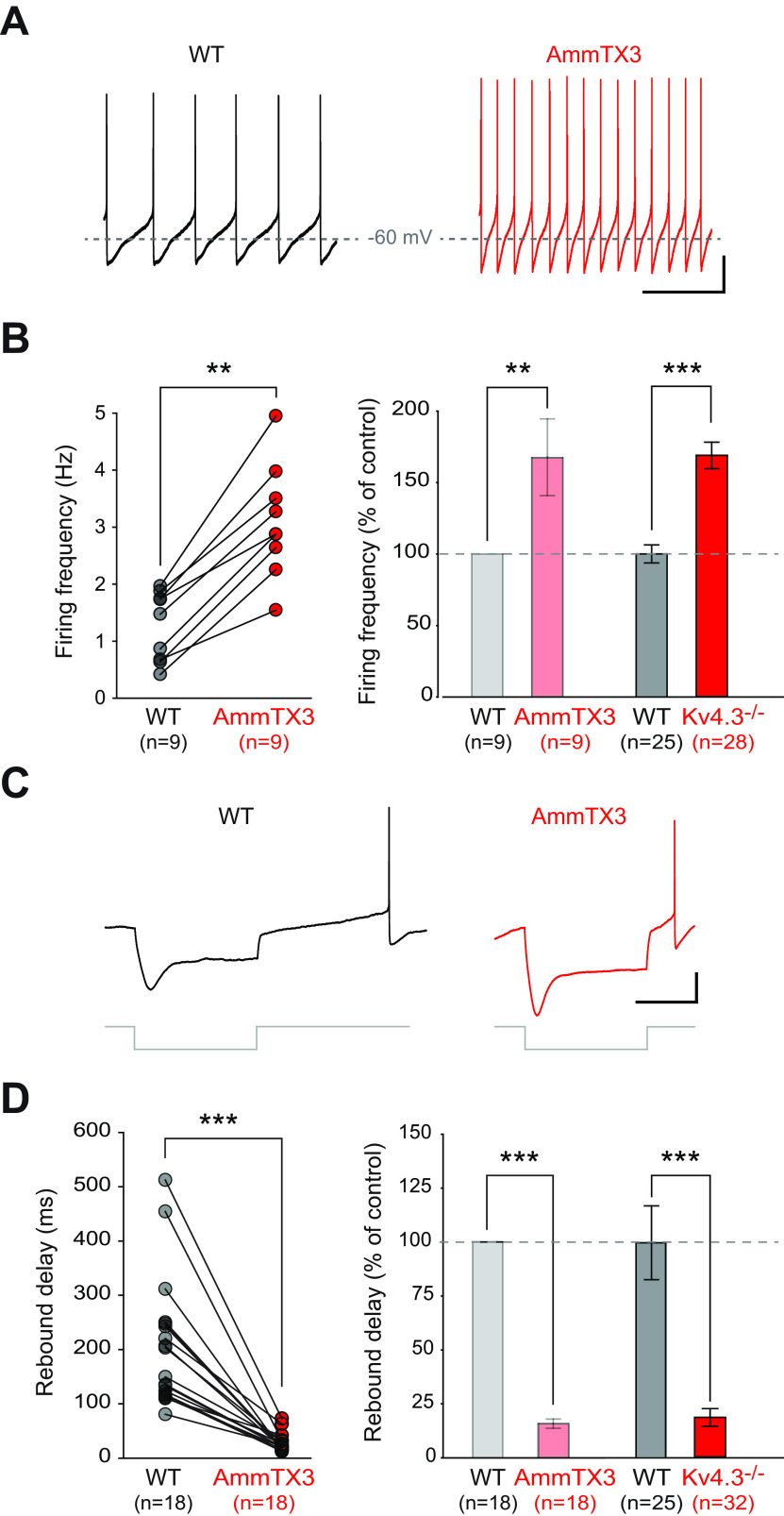
Comparing the alterations in electrophysiological phenotype after acute blockade of Kv4 channels with the Kv4.3−/− mouse model. ***A***, Current-clamp recordings showing the spontaneous pattern of activity of a WT SNc DA neuron in control condition (black trace, left) and after AmmTX3 application (red trace, right). ***B***, left, Line and scatter plot showing the change in spontaneous firing frequency induced by AmmTX3 application in individual WT SNc DA neurons. Right, Bar plot comparing the average change in spontaneous firing frequency after AmmTX3 application (left, light colors) or Kv4.3 channel deletion (right, dark colors). ***C***, Current-clamp recordings showing the voltage response of a WT SNc DA neuron to a hyperpolarizing current step (bottom gray traces) in control condition (left, black trace) and after AmmTX3 application (right, red trace). ***D***, left, Line and scatter plot showing the change in rebound delay induced by AmmTX3 application in individual WT SNc DA neurons. Right, Bar plot showing the average change in rebound delay after AmmTX3 application (left, light colors) or Kv4.3 channel deletion (right, dark colors); **p < 0.01, ***p < 0.001. Scale bars: 1 s (***A***, horizontal), 20 mV (***A***, vertical), –60 mV (***A***, horizontal gray dotted lines), 500 ms (***C***, horizontal), 20 mV (***C***, vertical).

Genetic deletion of Kv4.2 channels in cortical pyramidal neurons is associated with compensatory modifications in a delayed rectifier-like (I_KDR_-like) potassium current (Nerbonne et al., 2008). Moreover, I_H_ and I_A_ have complementary influences on postinhibitory firing in SNc DA neurons (Amendola et al., 2012; Tarfa et al., 2017). In order to reveal putative homeostatic compensations of Kv4.3 deletion in SNc DA neurons, we first performed a series of current-clamp recordings on a subset of neurons (*n* = 18 for WT, *n* = 32 for Kv4.3−/−) to compare the effect of acutely blocking Kv4 channels using the scorpion toxin AmmTX3 (Vacher et al., 2002) to the changes observed in the Kv4.3−/− mouse. Consistent with previous reports (Amendola et al., 2012; Tarfa et al., 2017), AmmTX3 strongly increased pacemaking frequency and dramatically reduced postinhibitory rebound delay (Fig. 6). Most interestingly though, the magnitude of the effects of the toxin was very similar to that observed in the Kv4.3−/− neurons: firing frequency was increased by ∼68% in both conditions (Fig. 6*B*), while rebound delay was decreased by 87% after AmmTX3 and by 82% in Kv4.3−/− mice (Fig. 6*D*). Consistent with the data presented earlier, these results strongly suggest that the Kv4-mediated A-type current is virtually completely abolished in Kv4.3−/− SNc DA neurons and that its loss is not compensated by changes in other Kv channels (and associated currents).

**Figure 7.**
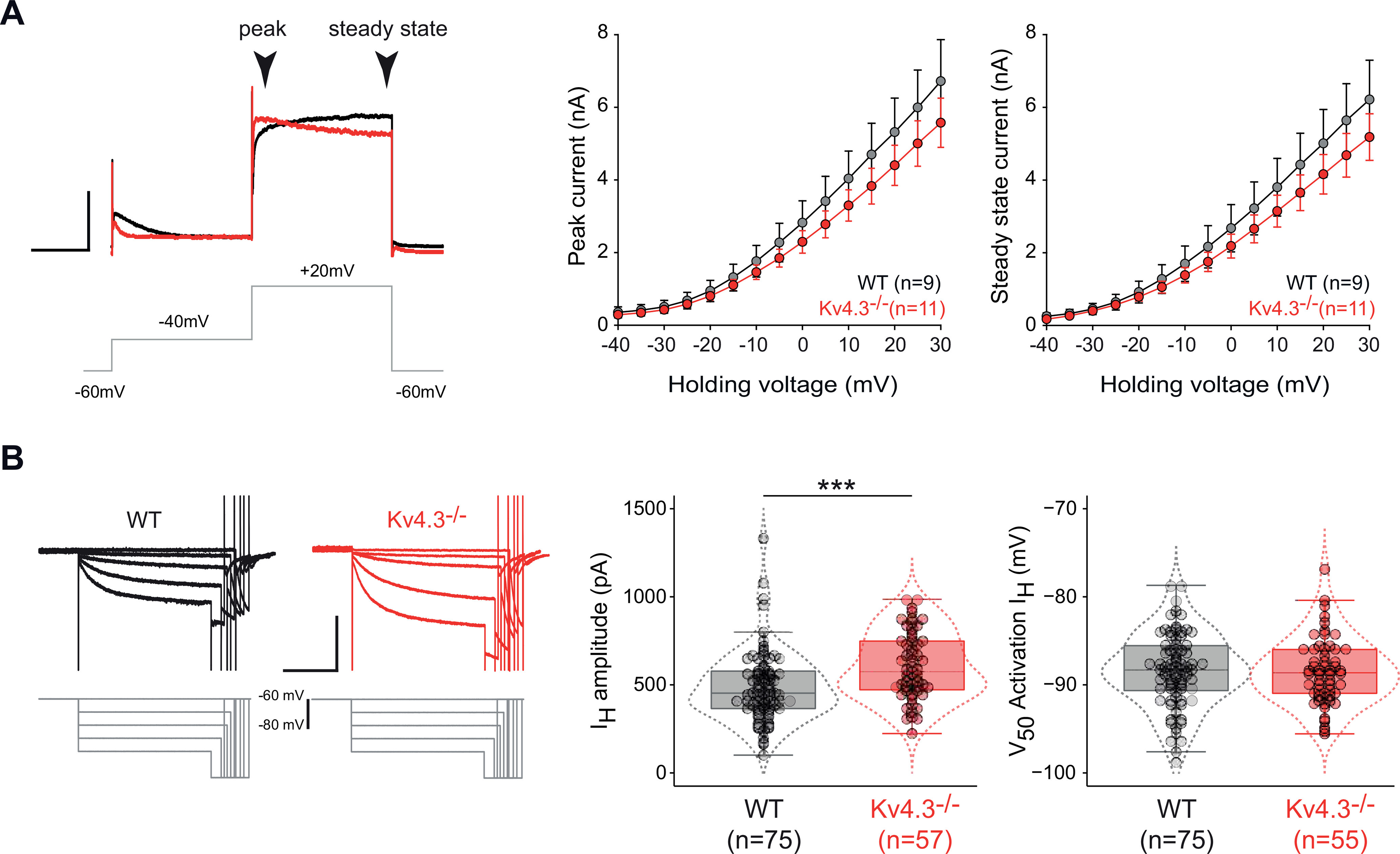
Absence of compensatory changes in delayed rectifier and I_H_ currents in the Kv4.3−/− SNc DA neurons. ***A***, Properties of the delayed rectifier potassium current (I_KDR_) in SNc DA neurons in WT and Kv4.3−/− mice. Left, Voltage-clamp recordings of I_KDR_ obtained in a WT (black trace) and a Kv4.3−/− mouse (red trace) in response to a voltage step to +20 mV (gray trace). The peak and steady-state components of I_KDR_ are indicated by arrowheads. Right, Line and scatter plots representing the average current–voltage relationships of the peak (left) and steady-state I_KDR_ (right) obtained from WT (gray dots) and Kv4.3−/− SNc DA neurons (red dots). ***B***, Properties of I_H_ in SNc DA neurons in WT and Kv4.3−/− mice. Left, Voltage-clamp recordings of I_H_ obtained in a WT (black traces) and a Kv4.3−/− mouse (red traces) in response to increasingly hyperpolarized voltage steps. Right, Box and whisker plot showing the distribution of values for I_H_ amplitude (left) and voltage sensitivity (right) in WT and Kv4.3−/− SNc DA neurons; ***p < 0.001. Dotted lines in the box and whisker plots indicate the distribution of data (violin plots). Scale bars: 200 ms (***A***, horizontal), 1 nA (***A***, vertical), 2 s (***B***, horizontal), and 500 pA (***B***, vertical).

We then used voltage-clamp recordings to directly assess whether a decrease in I_A_ could be compensated by a parallel decrease in I_H_ or a compensatory increase in I_KDR_ (Nerbonne et al., 2008; Fig. 7). Unlike what has been described in cortical neurons following Kv4.2 deletion, I_KDR_ was not modified in Kv4.3−/− SNc DA neurons (Fig. 7*A*). I_H_ was found to be slightly larger in Kv4.3−/− SNc DA neurons (Fig. 7*B*; Table 2), but its voltage sensitivity was unchanged. Altogether, the voltage-clamp recordings of I_A_, I_KDR_ and I_H_ and the AR Kv4.2 immunostaining suggest that Kv4.3 loss is not compensated by changes in expression and/or function of functionally-overlapping channels. These data provide a biophysical explanation for the observation made earlier that the acute blockade of Kv4 channels produces an electrophysiological phenotype qualitatively and quantitatively virtually identical to the Kv4.3 genetic deletion (Fig. 6).

### Bridging the gap between biophysical changes in I_A_ and I_H_ and variation in electrophysiological phenotype

**Figure 8.**
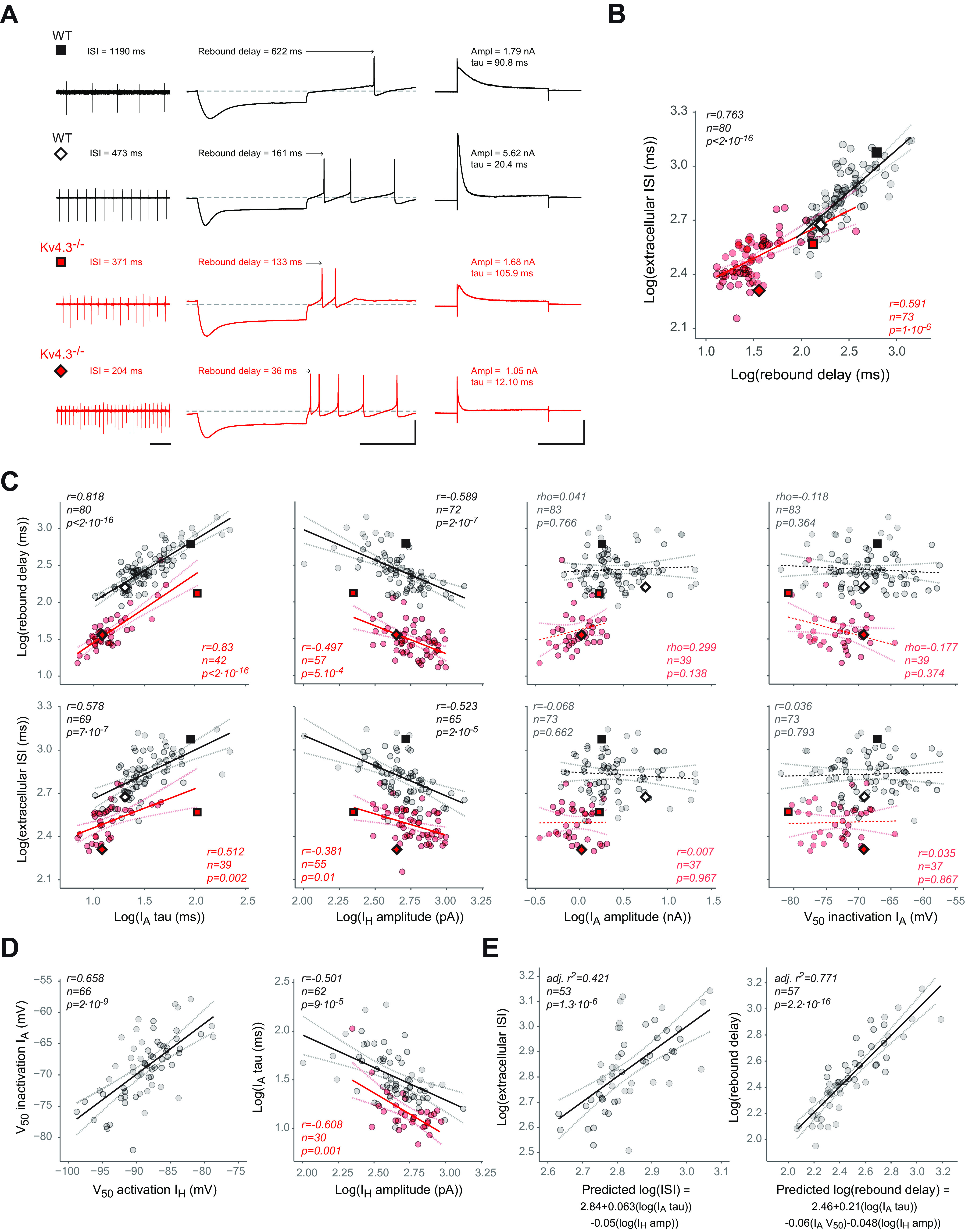
Linking biophysical changes in I_A_ to changes in electrophysiological phenotype in WT and Kv4.3−/− SNc DA neurons. ***A***, Recordings representative of the variation in spontaneous activity, postinhibitory rebound and I_A_ in WT and Kv4.3−/− SNc DA neurons. Left, Cell-attached voltage-clamp recordings of spontaneous pacemaking activity in two WT (black traces) and two Kv4.3−/− neurons (red traces). The value of the average ISI is indicated above the trace. Middle, Current-clamp recordings of the postinhibitory rebound obtained in the same neurons. The value of rebound delay is indicated above the trace. Right, I_A_ voltage-clamp recordings obtained at –40 mV in the same neurons. The values of I_A_ amplitude and tau are indicated above the trace. ***B***, Scatter plot showing the significant positive correlation between extracellularly recorded ISI and rebound delay observed in WT (gray dots) and Kv4.3−/− SNc DA neurons (red dots). The plain black and red lines correspond to the linear regression of the data (*r*, *n*, and *p* values are shown on the graph), while the gray and pink dotted lines indicate the regression confidence intervals. The diamond and square symbols correspond to the recordings presented in panel ***A***. ***C***, Scatter plots showing the relationships between biophysical variables and neuronal output. Correlations between I_A_ tau, I_H_ amplitude, I_A_ amplitude or I_A_ V_50_ (from left to right) and rebound delay or extracellular ISI (from top to bottom) were tested in both WT (gray dots) and Kv4.3−/− neurons (red dots). Please note that only I_A_ tau and I_H_ amplitude were significantly correlated with both ISI and rebound delay in WT and Kv4.3−/− neurons. The plain black and red lines correspond to the linear regression of the data (*r*/ρ, *n*, and *p* values are shown on the graph), while the gray and pink dotted lines indicate the regression confidence intervals. Dashed black and red lines indicate non-significant correlations. The diamond and square symbols correspond to the recordings presented in panel ***A***. ***D***, Scatter plots showing the significant correlations between I_A_ and I_H_ properties. Left, Scatter plot showing the positive correlation between I_A_ inactivation V_50_ and I_H_ activation V_50_ in WT neurons. Right, Scatter plot showing the negative correlation between I_A_ tau and I_H_ amplitude observed in both WT (gray dots) and Kv4.3−/− neurons (red dots). The plain black and red lines correspond to the linear regression of the data (*r*/ρ, *n*, and *p* values are shown on the graph), while the gray and pink dotted lines indicate the regression confidence intervals. ***E***, left, Scatter plot showing the multiple linear regression of extracellular ISI versus I_A_ tau and I_H_ amplitude (predicted ISI) in WT neurons. Right, Scatter plot showing the multiple linear regression of rebound delay versus I_A_ tau, I_A_ inactivation V_50_, and I_H_ amplitude (predicted rebound delay) in WT neurons. The corresponding equations are indicated below the *x*-axis of each graph. The plain black lines correspond to the linear regression of the data (*r*, *n*, and *p* values are shown on the graph), while the gray dotted lines indicate the regression confidence intervals. Scale bars: 1 s (***A***, left horizontal), 500 ms (***A***, middle horizontal), 50 mV (***A***, vertical), 250 ms (***A***, right horizontal), and 2 nA (***A***, right vertical).

We then decided to investigate whether the cell-to-cell variations in I_A_ biophysical properties in WT and Kv4.3−/− SNc DA neurons were predictive of variations in electrophysiological phenotype (Fig. 8). We first looked at potential correlations between firing parameters. As already presented in Figure 2, the most significant alterations in firing observed in Kv4.3−/− SNc DA neurons are a strong increase in spontaneous firing frequency (strong decrease in the extracellularly-measured ISI) and a strong decrease in postinhibitory rebound delay. We found that extracellular ISI and rebound delay (log transformed) were strongly positively correlated with each other in both WT and Kv4.3−/− neurons (Fig. 8*B*), although the slope of this relationship seemed slightly different between the two genotypes. We therefore tried to determine whether specific I_A_ biophysical properties were better predictors of these variations in ISI or rebound delay (Fig. 8*C*). We also analyzed the relationship between I_H_ properties and ISI or rebound delay. Out of the 5 biophysical parameters analyzed (I_A_ tau, I_H_ amplitude, I_A_ amplitude, I_A_ inactivation V_50_, I_H_ activation V_50_), only two parameters were significantly correlated with ISI or rebound delay: I_A_ tau and I_H_ amplitude (log transformed) were positively and negatively correlated, respectively, with both ISI and rebound delay in both WT and Kv4.3−/− neurons (Fig. 8*C*). Surprisingly, neither I_A_ amplitude (measured at –40 mV) nor its voltage dependence (inactivation V_50_) were predictive of variations in ISI or rebound delay (Fig. 8*C*). I_H_ activation V_50_ was also unable to predict variations in these firing parameters (data not shown). In addition, reminiscent of the observation made in rat neurons (Amendola et al., 2012), I_H_ activation and I_A_ inactivation V_50_s were found to be positively correlated (Fig. 8*D*). I_A_ tau and I_H_ amplitude were also found to be negatively correlated (Fig. 8*D*). Based on these observations, we then tested whether combining several of these 5 biophysical parameters could improve the prediction of ISI or rebound delay using multiple linear regression (Fig. 8*E*). We first standardized these parameters (subtracting the mean and dividing by the SD), and then looked for the best subset of variables predictive of ISI or rebound delay. While ISI was best predicted by a multiple linear regression involving only I_A_ tau and I_H_ amplitude, rebound delay was better predicted when I_A_ tau, I_H_ amplitude, and I_A_ inactivation V_50_ were included in the linear regression (Fig. 8*E*). Rebound delay prediction was much more accurate than ISI prediction (*r*^2^ = 0.771 compared with *r*^2^ = 0.421). Based on the scaling factors given by the multiple linear regression, it is important to note that both ISI and rebound delay in real neurons seem to be most sensitive to variations in I_A_ tau.

### Influence of I_A_ biophysical properties on SNc DA neuron firing

**Figure 9.**
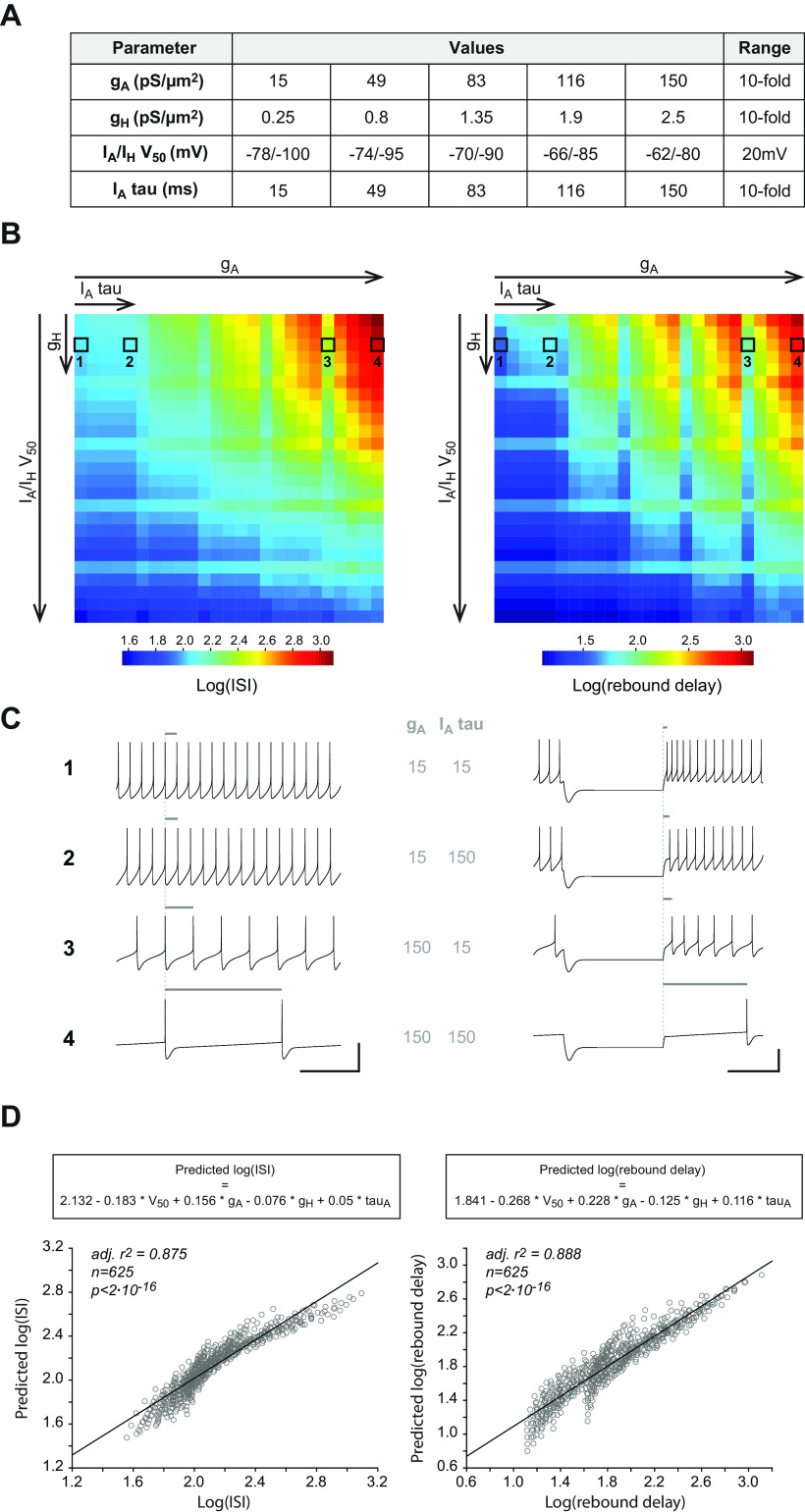
Modeling the effect of the biophysical properties of I_A_ and I_H_ on spontaneous activity and postinhibitory rebound. ***A***, Table presenting the five values tested for each biophysical parameter (g_A_, g_H_, I_A_/I_H_ V_50_, and I_A_ inactivation tau) in the multicompartment model. Each property was varied independently, leading to 625 (5^4^) versions of the model. ***B***, Dimensionally stacked heatmaps showing the variation in ISI [displayed as log(ISI); left] and in postinhibitory rebound delay [displayed as log(rebound delay); right] as a function of g_A_ (*x*-axis, big scale), I_A_/I_H_ V_50_ (*y*-axis, big scale), I_A_ tau (*x*-axis, small scale), and g_H_ (*y*-axis, small scale). ***C***, Example traces of spontaneous pacemaking (left) and postinhibitory rebound (right) obtained for the minimal and maximal values of g_A_ and I_A_ tau (corresponding to the four squares surrounded in the panel ***B*** heatmaps). The gray horizontal bars above the traces help visualize the change in ISI (left) or rebound delay (right) as a function of changes in g_A_ and I_A_ tau. ***D***, Multiple linear regression reveals the relative contribution of each biophysical property to ISI (left) or rebound delay variation (right). The scatter plots show the relationship between the measured values of log(ISI) or log(rebound delay) and the values predicted using a linear combination of the four biophysical variables listed in panel ***A*** (the corresponding equations are shown above each graph). Scale bars: 500 ms (***C***, horizontal) and 50 mV (***C***, vertical).

Many conductances other than I_A_ and I_H_ may vary in expression level and biophysical properties from neuron to neuron, potentially compensating or enhancing the effect of variations in the properties of these two currents on firing (Gentet and Williams, 2007; Puopolo et al., 2007; Seutin and Engel, 2010; Amendola et al., 2012; Philippart et al., 2016; Moubarak et al., 2019). In order to isolate the influence of specific biophysical properties of I_A_ and I_H_ on SNc DA neuron activity, we used a realistic multicompartment Hodgkin–Huxley model of rat SNc DA neurons (Moubarak et al., 2019). Based on measurements obtained by different groups (Liss et al., 2001; Gentet and Williams, 2007; Amendola et al., 2012; Tarfa et al., 2017), each of the biophysical properties of I_A_ (maximal conductance g_A_, voltage-dependence I_A_ V_50_, inactivation rate I_A_ tau) and I_H_ maximal conductance (g_H_) were varied over a 10-fold range (20-mV range for the voltage dependence) using five equidistributed values (Fig. 9*A*). I_A_ and I_H_ voltage dependences were forced to co-vary in the model, based on our previous observations (Fig. 8*D*) (Amendola et al., 2012). Using a sample of 22 realistic models and five independently-varying values for each biophysical property, a database of 13,750 models (22 × 5^4^) was generated (Fig. 9). The average ISI during spontaneous activity and the postinhibitory firing delay in response to a hyperpolarizing pulse (rebound delay) were measured for each model and their average values were calculated for each combination of values of the four biophysical parameters (*n* = 625). Dimensional stacking (Taylor et al., 2006) was then used to represent the influence of the four biophysical parameters on these electrophysiological features in two-dimensional heatmaps, allowing us to visually determine which parameters were most critical in controlling ISI and rebound delay (Fig. 9*B*,*C*): g_A_ and I_A_/I_H_ V_50_ strongly modulated both ISI and rebound delay while I_A_ tau and g_H_ had a weaker influence on these firing properties. To quantify the contribution of each biophysical parameter to ISI and rebound delay variations, we then used the same strategy already presented in Figure 8*E* for experimental measurements: standardized parameters were used to run multiple linear regression against ISI or rebound delay (Fig. 9*D*). Consistent with the visualization provided in Figure 9*B*, this sensitivity analysis revealed that the influence of g_A_ and I_A_/I_H_ V_50_ on ISI and rebound delay was two to three times stronger than that of g_H_ and I_A_ tau. While the results of this computational modeling are consistent with the general influence of I_A_ and I_H_ reported in these neurons (Liss et al., 2001; Seutin et al., 2001; Neuhoff et al., 2002; Puopolo et al., 2007; Amendola et al., 2012; Gantz et al., 2018), they reveal specific effects of I_A_ biophysical properties contrasting with the correlations identified in our experimental data.

## Discussion

The current study provides important elements regarding the identity of the ion channels underlying I_A_ in SNc DA neurons and the relative influence of specific biophysical parameters of I_A_ (voltage dependence, gating kinetics, maximal conductance) on neuronal output. In particular, in contrast with previous studies (Serôdio and Rudy, 1998; Liss et al., 2001; Dufour et al., 2014a), we show that the Kv4.2 subunit is expressed in SNc DA neurons, although its functional influence appears negligible in most neurons, because of a very low level of expression. Despite Kv4.2 presence, we show that the constitutive loss of Kv4.3 is not compensated by complementary intrinsic conductances (Kv4.2-mediated I_A_, I_KDR_, I_H_). In addition, while previous studies (Liss et al., 2001; Putzier et al., 2009b) and the computational modeling performed here suggest a strong role of I_A_ conductance in controlling firing frequency, we demonstrate that I_A_ gating kinetics appear as the major determinant of both pacemaking frequency and postinhibitory rebound delay. Our results also highlight the functional complementarity and correlation of biophysical properties of I_A_ and I_H_ in these neurons.

### Kv4.2 is expressed in SNc DA neurons

One of the important results of the present work is the demonstration that Kv4.2 is expressed in mouse SNc DA neurons. So far, it was thought that only Kv4.3 was expressed and entirely responsible for the large A-type current observed in these neurons (Liss and Roeper, 2008; Gantz et al., 2018). Our results however unambiguously demonstrate that Kv4.2 is expressed by SNc DA neurons. First, in the absence of Kv4.1 (Liss et al., 2001; Ding et al., 2011), the presence of an AmmTX3-sensitive residual A-type current in the Kv4.3−/− neurons is only compatible with the expression of Kv4.2. While the Kv4.2-mediated residual I_A_ is very small and fast in most neurons, in 12% of the voltage clamp-recorded neurons it is large enough to confer a WT phenotype (see Figs. 5, 9). Although this residual I_A_ current is much faster than its WT counterpart in most neurons, it still influences firing, particularly rebound delay, as suggested by the highly significant correlation between I_A_ tau and rebound delay. Second, AR immunohistochemistry confirmed that a Kv4.2 staining is observed in a minority (∼5%) of SNc DA neurons in both Kv4.3−/− and WT mice. While the percentage of cells displaying a “high” expression of Kv4.2 is too small to allow a combined voltage-clamp/AR immunohistochemistry approach, the similarity in the proportion of AmmTX3-sensitive “large residual” I_A_ (∼12%) and Kv4.2-positive neurons (∼5%, not statistically different) strongly suggests that Kv4.2 is responsible for a large I_A_ in a minority of SNc DA neurons. The presence of a small AmmTX3-sensitive residual I_A_ in the rest of the Kv4.3−/− SNc DA neurons suggests that Kv4.2 is expressed and functional in all SNc DA neurons, although its influence might be minor in the presence of Kv4.3. The fact that the residual A-type current in Kv4.3−/− neurons inactivates faster than its WT counterpart is also consistent with the reported differences in inactivation kinetics of Kv4.2 and Kv4.3 (Serôdio et al., 1994, 1996). Altogether, these results suggest that, in contrast to the widely accepted view, a small subpopulation of SNc DA neurons (∼5–10%) display an A-type current likely mediated by both Kv4.3 and Kv4.2 channels. Interestingly, these two subunits are very similar and co-immunoprecipitate from mouse brain lysates (Marionneau et al., 2009), suggesting that they could form heteromeric I_A_ channels. At this point however, it is difficult to determine whether the Kv4.2-positive neurons in WT mice express Kv4.3/Kv4.2 heteromers or distinct Kv4.3 and Kv4.2 homomers.

### Lack of homeostatic compensation in Kv4.3−/− SNc DA neurons

Intriguingly, these results not only suggest that Kv4.2 expression is very low in most SNc DA neurons but also that it is not modified in the Kv4.3−/− SNc DA neurons. The structural and functional similarities between Kv4.3 and Kv4.2 means that in theory Kv4.2 channels should be able to compensate for the loss of Kv4.3. Despite this, the Kv4.2 pattern of expression is not modified in Kv4.3−/− SNc DA neurons. This is reminiscent of previous studies performed on the Kv4.2−/− mouse demonstrating that Kv4.3 expression pattern, assessed by western-blot or immunohistochemistry, is not modified following Kv4.2 loss (Menegola and Trimmer, 2006; Nerbonne et al., 2008). In addition, other currents (such as I_KDR_ or I_H_) also appear to not be regulated in a compensatory direction in Kv4.3−/− SNc DA neurons. The current-clamp comparison between the acute blockade of Kv4 channels in WT neurons and the Kv4.3−/− neurons also supports this idea of a lack of compensatory modifications in functionally-overlapping currents. This is surprising in the light of the results obtained on neonatal cortical pyramidal cells (Nerbonne et al., 2008), where Kv4.2 genetic deletion is almost “fully” compensated by an increase in sustained potassium currents. To explain this difference, we may hypothesize that, although the alteration in electrophysiological phenotype observed in Kv4.3−/− neurons is striking (see Fig. 3 in particular), the change in calcium dynamics associated with the elevated spontaneous activity may not be sufficient to trigger homeostatic regulatory mechanisms (O’Leary et al., 2014). Alternatively, modifications in the properties of incoming excitatory or inhibitory synaptic inputs (not analyzed in the current study) may compensate for the changes in intrinsic activity reported here, such that the overall *in vivo* activity of the SNc network is maintained in Kv4.3−/− animals. However, behavioral alterations suggest that the change in firing of SNc DA neurons is not totally compensated at the network level. Kv4.3−/− mice were more active than WT littermates in exploring the environment (increased rearing behavior) and could not adjust their motor control over sessions on the rotarod. This suggests that the changes in midbrain DA neuron tonic firing observed in the Kv4.3−/− SNc DA neurons might only be revealed when animals are exploring their environment or challenged in a motor learning task.

### I_A_ gating kinetics play a central role in SNc DA neuron output

The use of I_A_ voltage-clamp measurements and current-clamp recordings on a large number of neurons in WT and Kv4.3−/− mice allowed us to determine the impact of cell-to-cell variations in I_A_ biophysical properties on spontaneous activity and rebound delay. Interestingly, while realistic multicompartment modeling suggested that I_A_ maximal conductance and voltage dependence were the two factors most strongly influencing these electrophysiological features, our recordings revealed that I_A_ inactivation rate was the dominant factor defining pacemaking frequency and rebound delay (Fig. 9*E*). I_H_ amplitude (proportional to I_H_ maximal conductance in our measurements) was also found to play an important role in real neurons, while its influence was minor in the model. These differences may be explained by several factors. First, the database approach used for our simulations implies that all the tested biophysical properties are varied independently (except for the strict correlation applied to I_A_ and I_H_ V_50_s). While the independence between these biophysical parameters allowed us to precisely quantify the sensitivity of spontaneous activity and rebound delay to each parameter, it does not correspond to the observations made in real neurons: for instance, I_H_ amplitude and I_A_ inactivation rate are negatively correlated, i.e., not independent from each other. On the other hand, reminiscent of a previous study performed on rat neurons (Amendola et al., 2012), we demonstrated that I_A_ and I_H_ V_50_s are also positively correlated in WT mouse neurons. However, this correlation is much lower than the one applied in the model (*r* = 0.658, *r*^2^ = 0.43 compared with *r*^2^ = 1) and this difference may explain why this biophysical parameter appears as one of the most efficient in modulating firing in the model. Thus, the differences in independence of the biophysical properties may partly explain why the model and the experimental observations give different answers. Another factor may explain why I_A_ maximal conductance has a strong effect on firing in the model, but not in real neurons. In order to isolate the effect of I_A_ and I_H_ biophysical properties on firing, all other conductances included in the model were held at fixed values. However, every ion current displays significant cell-to-cell variations in its properties (gating, conductance density) in a same neuronal population (Swensen and Bean, 2005; Schulz et al., 2006; Amendola et al., 2012; Moubarak et al., 2019). If happening at random, these variations in other currents would most likely dampen the effect of the variations in I_A_ or I_H_ specific properties on firing. In fact, we demonstrated in a previous study (Tapia et al., 2018) that the level of expression of Kv4.3 (at the mRNA level) in midbrain DA neurons co-varies with the expression levels of multiple somatodendritic ion channels, including Nav1.2, SK3, and GIRK2. If this co-variation is retained at the protein level, it would mean that cell-to-cell variations in I_A_ maximal conductance occur in parallel with variations in density of other ion channels. Whether correlated or not, cell-to-cell variations in other conductances may thus explain why I_A_ amplitude does not predict pacemaking and rebound delay in real neurons, and why I_A_ inactivation rate appears as the main predictor of these electrophysiological features. In contrast with our findings, the results obtained by Liss et al. (2001) suggested that Kv4.3 expression level and channel density predicted pacemaking frequency in mouse neurons. Interestingly, it is noteworthy that I_A_ inactivation rate showed restricted cell-to-cell variations in their recordings (2-fold range). On the other hand, I_A_ charge density showed a 10-fold range of variation, suggesting that most of the variation in I_A_ function was because of variations in I_A_ maximal conductance (Liss et al., 2001). In our recordings however, the levels of variability observed for I_A_ (and I_H_) biophysical properties were rather similar, which led us to apply a 10-fold range to each parameter in our model. Thus, we postulate that the differences in our conclusions may be essentially related to differences in the cell-to-cell variability range of I_A_ and I_H_ biophysical parameters recorded in our samples.

### Functional complementarity and co-regulation of I_A_ and I_H_ in SNc DA neurons

While our results confirm the well-established influence of I_A_ on SNc DA neuron firing (Liss et al., 2001; Gentet and Williams, 2007; Putzier et al., 2009b; Amendola et al., 2012; Tarfa et al., 2017), they also emphasize the functional complementarity between I_A_ and I_H_ in these neurons, and reinforce the idea that the channels underlying these currents are co-regulated (Amendola et al., 2012). Indeed, we confirm that I_A_ and I_H_ voltage dependences are positively correlated and show that I_H_ amplitude and I_A_ inactivation rate are negatively correlated. While we do not have a mechanistic explanation for this latter correlation, these results are reminiscent of the observations made by Tarfa and colleagues on nigrostriatal and mesoaccumbal DA neurons (Tarfa et al., 2017). At the functional level, our results demonstrate that these two parameters are the main predictors of the cell-to-cell variations in pacemaking frequency and rebound delay, reinforcing the idea that I_A_ and I_H_ function as a complementary pair of currents tightly controlling postinhibitory rebound delay in SNc DA neurons (*r*^2^ = 0.77). The fact that pacemaking rate is not as accurately predicted by I_A_ and I_H_ properties (*r*^2^ = 0.42) is consistent with the documented role of many other conductances and morphologic parameters in defining this firing feature (Nedergaard and Greenfield, 1992; Wilson and Callaway, 2000; Wolfart et al., 2001; Liss et al., 2005; Puopolo et al., 2007; Putzier et al., 2009a; Gantz et al., 2018; Moubarak et al., 2019).

## Synthesis

Reviewing Editor: Matthew Grubb, King's College London Decisions are customarily a result of the Reviewing Editor and the peer reviewers coming together and discussing their recommendations until a consensus is reached. When revisions are invited, a fact-based synthesis statement explaining their decision and outlining what is needed to prepare a revision will be listed below. The following reviewer(s) agreed to reveal their identity: NONE. Note: If this manuscript was transferred from JNeurosci and a decision was made to accept the manuscript without peer review, a brief statement to this effect will instead be what is listed below.

Synthesis:

Both reviewers approve the revisions and support publication of the manuscript as is.

Reviewers' comments:

R1: The authors have addressed my comments on the original version of this manuscript in revising the text and in written replies. I am still of the opinion that neither the behavior nor the modeling adds much to this paper (and the three sections of the manuscript remain rather disconnected). The authors, however, have clearly articulated their reasons for retaining both components and I don't see any reason to debate the points further.

R2: This manuscript was transferred from the Journal of Neuroscience where I was a reviewer for the previous version. The authors have adequately addressed all of my concerns in the current manuscript. This is a rigorous, high quality study that makes an important contribution to field. Congratulations to the all of the authors!
